# RASPD+: Fast Protein-Ligand Binding Free Energy Prediction Using Simplified Physicochemical Features

**DOI:** 10.3389/fmolb.2020.601065

**Published:** 2020-12-17

**Authors:** Stefan Holderbach, Lukas Adam, B. Jayaram, Rebecca C. Wade, Goutam Mukherjee

**Affiliations:** ^1^Molecular and Cellular Modelling Group, Heidelberg Institute of Theoretical Studies, Heidelberg, Germany; ^2^Institute of Pharmacy and Molecular Biotechnology (IPMB), Heidelberg University, Heidelberg, Germany; ^3^Supercomputing Facility for Bioinformatics & Computational Biology, Department of Chemistry, Kusuma School of Biological Sciences, Indian Institute of Technology Delhi, New Delhi, India; ^4^Center for Molecular Biology (ZMBH), DKFZ-ZMBH Alliance, Heidelberg University, Heidelberg, Germany; ^5^Interdisciplinary Center for Scientific Computing (IWR), Heidelberg University, Heidelberg, Germany

**Keywords:** structure based drug design, virtual screening, physicochemical molecular descriptors, machine learning, protein-ligand complex, binding free energy

## Abstract

The virtual screening of large numbers of compounds against target protein binding sites has become an integral component of drug discovery workflows. This screening is often done by computationally docking ligands into a protein binding site of interest, but this has the drawback of a large number of poses that must be evaluated to obtain accurate estimates of protein-ligand binding affinity. We here introduce a fast pre-filtering method for ligand prioritization that is based on a set of machine learning models and uses simple pose-invariant physicochemical descriptors of the ligands and the protein binding pocket. Our method, Rapid Screening with Physicochemical Descriptors + machine learning (RASPD+), is trained on PDBbind data and achieves a regression performance that is better than that of the original RASPD method and traditional scoring functions on a range of different test sets without the need for generating ligand poses. Additionally, we use RASPD+ to identify molecular features important for binding affinity and assess the ability of RASPD+ to enrich active molecules from decoys.

**Graphical Abstract:**
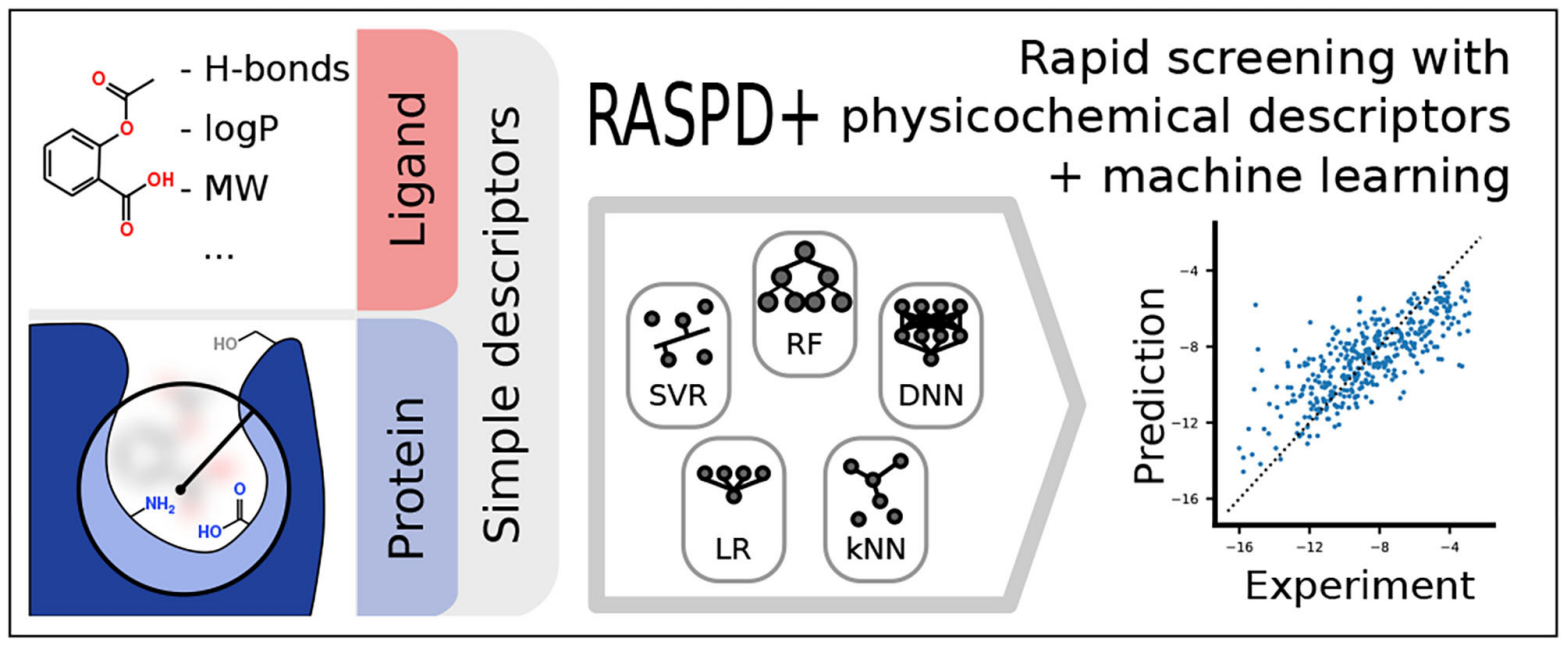
Overview of the overall workflow of RASPD+ (Rapid Screening with Physicochemical Descriptors + machine learning).

## 1. Introduction

Virtual screening to assess *in silico* the binding of candidate ligands to a target protein is a key component of structure-based drug design procedures (Torres et al., 2019; Wang et al., 2020). Typically, screening is done by docking the ligands at many different positions or poses in the three-dimensional structure of the target protein. At every position, a scoring function is evaluated to approximate the binding-free energy, and this is used to rank the binding poses and different candidate ligands for their ability to bind to the target protein. While correct docking poses are frequently generated, scoring functions often lack the accuracy necessary to correctly rank poses or ligands (Li et al., 2019). Docking procedures are therefore frequently supplemented by methods employing molecular dynamics simulations with the aim of computing more accurate binding affinities. However, both docking and molecular dynamics simulations often fail to provide predictions of binding free energy at the level of accuracy desired. Furthermore, they are demanding in terms of computational effort and expertise (Willems et al., 2020). There is therefore a need for quick approaches with robust predictive scoring functions to facilitate the screening and prioritization of large libraries of compounds prior to applying docking and simulation methods.

**Figure 1:**
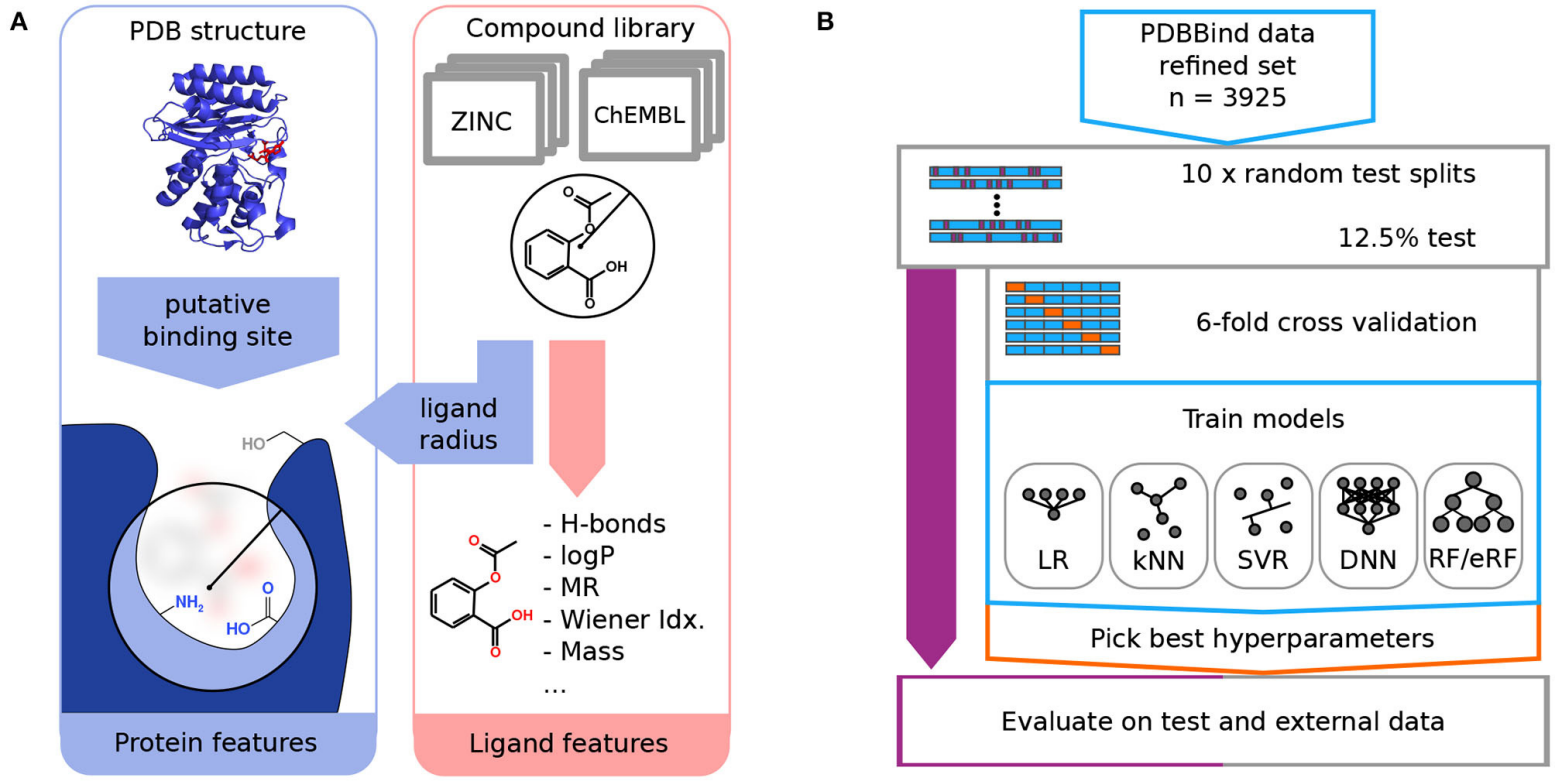
The computational workflow of RASPD+ comprises featurization **(A)** and the training and evaluation of machine learning models **(B)**. **(A)** For each ligand molecule, simple physicochemical descriptors are computed based on atomic contributions. Information about the target protein is gathered within a sphere around a putative binding position whose size is determined by the radius of the ligand. For residues within this sphere, similar descriptors are computed. **(B)** Data from the PDBbind refined set featurized in this way served as the training data in a nested cross-validation strategy. To compare linear regression (LR), k-nearest neighbors (kNN), support vector regression (SVR), neural network (DNN), random forest (RF), and extremely random forest (eRF) models, test sets were split off in 10 replicates in an outer loop. In the inner loop, six-fold cross-validation was used to select the best hyperparameters for the given model.

While the assessment of ligand properties, e.g., for drug-likeness (Lipinski et al., 2001), to filter ligand libraries is well established, we here address the need to filter and prioritize ligands based not only on ligand properties but also on the properties of the target protein. For this purpose, we previously developed a simple hybrid regression approach called RASPD (Rapid Screening with Physicochemical Descriptors) (Mukherjee and Jayaram, 2013). In this linear regression model, the binding-free energy Δ*G* was predicted using a minimal set of physicochemical descriptors for typical interactions. Hydrogen bonding was accounted for by counting potential donor and acceptor atoms. Van der Waals forces were approximated by the Wiener topology index (Wiener, 1947) and the molar refractivity, which describes the polarizability of a molecule (Ghose and Crippen, 1987). Additionally, the partition coefficient logP allowed for the estimation of the hydrophobic effect. While the descriptor values for the ligand are straightforward to compute, simplifying assumptions were made to obtain the physicochemical descriptors for the target protein. A sphere was centered on a known or assumed binding pocket position with a radius encompassing the maximum size of the ligand. This sphere was then used to select the amino acid residues for which descriptors were computed (Mukherjee and Jayaram, 2013) (Figure 1A).

However, the linear regression model used (Mukherjee and Jayaram, 2013) has limited abilities to capture complex feature interactions compared to non-linear models. Since RASPD was first developed, more high-quality data sets on protein-ligand complexes with associated binding-free energies have been made available (Liu et al., 2015; Gathiaka et al., 2016), and a large number of machine learning methods have been developed (Yang et al., 2019). Moreover, machine learning approaches have successfully been used to either replace (Gomes et al., 2017; Feinberg et al., 2018; Jiménez et al., 2018) or enhance (Pei et al., 2019; Boyles et al., 2020) the predictions of traditional scoring functions for protein-ligand binding.

We have thus developed RASPD+, which is a new tool that improves on the conceptual framework of the original RASPD method by using the following: (i) a set of diverse machine learning methods to derive an ensemble prediction, (ii) additional and more fine-grained descriptors for the target proteins, and (iii) larger training sets of newer protein-ligand binding data. We here describe the training, testing, and application of RASPD+. We demonstrate the capabilities of RASPD+ for binding free energy regression and compare its performance to established scoring functions. We also analyze the features contributing to the predictions to gain insights into the important features for binding affinity. Finally, we show that RASPD+ can enrich active molecules in tests with the Directory of Useful Decoys-Enhanced data set (DUD-E) (Mysinger et al., 2012).

## 2. Methods

The computational workflow and the training and validation procedure used for RASPD+ are illustrated in Figure 1.

### 2.1. Datasets

The PDBbind refined data set (release 2018) (Wang et al., 2004; Liu et al., 2015), containing 4,463 protein-ligand crystal structures and experimentally measured binding affinities, served as the initial data set. Although the PDBbind refined data set contains data on binding from different measurements reported in the literature and no experimental method was specified as a requirement for the data to be incorporated in PDBbind, it contains high-quality structures of non-covalent protein-ligand interactions with a resolution better than 2.5 Å and no steric clashes. The PDBbind refined data set is therefore extensively used as a benchmark set for protein-ligand binding affinity prediction (Liu et al., 2015). We thus obtained structural information about each protein in the data set, the position and structure of the ligand binding to it, and the corresponding binding constant. As we considered modeling the coordination of metal ions to be beyond the scope of our approach, the structures were filtered to exclude cases with metal ions within 2.1 Å of the ligand. Dissociation and inhibition constants and *IC*_50_ values were converted to binding-free energies using the following equation:

(1) $$\begin{array}{r} \Delta G=-RT\ln\;K\text{ where }K\in \left\{K_{d},K_{i},{IC}_{50}\right\}\text{ assuming}\\ \;T=298.15\;K \end{array}$$

This processing resulted in a set of 3,925 protein-ligand complexes for training, validation, and testing.

For further testing, the following previously published benchmark sets served as external test sets: The Community Structure-Activity Resource (CSAR) NRC-HiQ 2010 selection (Dunbar et al., 2011; Smith et al., 2011), data sets from the CSAR 2012 (Dunbar et al., 2013), and CSAR 2014 (Carlson et al., 2016) challenges, and a data set described by Wang et al. (2015).

The CSAR-NRC 2010 HiQ release (Dunbar et al., 2011; Smith et al., 2011) contains two sets of protein-ligand complexes, with 55 and 49 docked complexes, respectively, as well as information about experimental binding affinities.

Another set of binding-free energies and corresponding structures was assembled from the CSAR 2012 (Dunbar et al., 2013) and CSAR 2014 (Carlson et al., 2016) data sets that are now curated by the Drug Design Data Resource (D3R) (drugdesigndata.org) (Gathiaka et al., 2016). For this set, which we refer to as the D3R data set, we downloaded the data for the proteins urokinase, cyclin-dependent kinase 2 (CDK2), checkpoint kinase 1 (CHK1), MAP kinase 1 (ERK2), LpxC deacetylase (LpxC), spleen tyrosine kinase (SYK), tRNA (m1G37) methyltransferase (tRMD), heat shock protein 90 (HSP90), and a CDK2-Cyclin A complex. The SMILES strings of 1,271 active inhibitors of these proteins in the D3R data set were converted to 3D structures in PDB format using Open Babel (O'Boyle et al., 2011). For HSP90, we excluded 46 compounds that were all assigned the same Δ*G* of -5.860 kcal/mol as this value, likely represented a threshold value for the experimental measurements rather than the actual binding affinity of the ligands.

Wang et al. (2015) aggregated previous experimental results and PDB structures for 283 complexes of seven different proteins: beta-secretase (BACE), CDK2, induced myeloid leukemia cell differentiation protein (Mcl-1), p38 MAP kinase, protein-tyrosine phosphatase 1B (PTP1B), thrombin, and tyrosine kinase 2 (TYK2). For this set, protein structures were retrieved from the RCSB protein data bank (http://www.rcsb.org) and hydrogen atoms were added to the protein structures with the tleap module of AMBER 14 (Case et al., 2005). The structural data for inhibitors and experimental binding-free energies were obtained from the literature (Wilson et al., 2007; Baum et al., 2009; Goldstein et al., 2011; Cumming et al., 2012; Friberg et al., 2013; Liang et al., 2013a,b; Wang et al., 2013, 2015). This included additional ligands for Mcl-1 (Friberg et al., 2013) and TYK2 (Liang et al., 2013a,b) that were not used by Wang et al. (2015). The structures of the 283 inhibitors were redrawn and verified in the MOE software (Chemical Computing Group, Montreal, QC).

Further details on the source of structures and experimental binding affinities are given in Supplementary Table 1.

### 2.2. Generation of Molecular Descriptors

To model the non-covalent interactions, physicochemical molecular descriptors were computed using an improved pipeline based on that for the original RASPD procedure described in Mukherjee and Jayaram (2013) (Figure 1A). For each ligand, the molecular weight (here abbreviated as MASS), the number of hydrogen bond donors (D) and acceptors (A), an approximate octanol-water partition coefficient log P (logP) (Wildman and Crippen, 1999), the molar refractivity (MR) (Wildman and Crippen, 1999), and the Wiener topology index (W) (Wiener, 1947) were computed as described previously (Mukherjee and Jayaram, 2013). Based on the ligand position in the protein structure, the most likely interacting amino acid residues were selected using a sphere whose radius was derived from the maximum distance (*maxD*) between ligand atoms and the center of mass (Figure 1A). For the computation of the logP and MR descriptors, this sphere was extended by 0.9 Å over *maxD*, and residues were selected based on their center of mass. To count hydrogen bond donors and acceptors, a sphere extending 3 Å beyond *maxD* was used to select atoms. Details regarding the protein pocket selection procedure and the choice of the cut-off radii are given in Mukherjee and Jayaram (2013). To make the protein descriptors more fine grained than in the previous RASPD procedure, we computed molar refractivity and log P for aromatic and non-aromatic residues separately [PMR(Arom), PMR(Non-Arom), PlogP(Arom), PlogP(Non-Arom)]. Hydrogen bond donors were counted separately for the backbone amide group [PD(Amide-NH)] as well as for the following amino acid sets: Positively charged PD (K+R+HIP), neutral amino groups PD(K+N+Q), heteroaromatic donors PD (W+H), and hydroxyl-containing groups PD (T+S+Y+D+E). The number of hydrogen bond acceptors was determined for the backbone amide [PA(Amide-O)] and the following sets: negatively charged PA (D+E), neutral non-aromatic PA (N+Q+T+S+D-H+E-H), and aromatic acceptors PA (Y+H). The individual protein residue-derived descriptors were scaled by the ligand *maxD*. Additionally, the volume of the protein pocket (PVol) was computed using tools from the TRAPP software suite (Kokh et al., 2013; Yuan et al., 2020). In total, therefore, six ligand and 14 protein descriptors were computed per ligand-protein complex.

### 2.3. General Strategy for Training and Testing

To obtain a robust estimate of performance on the PDBbind data set as well as the test sets, a nested cross-validation strategy was used (Figure 1B). For 10 replicates, the PDBbind refined set was split into a test set covering 12.5% of the data and a set for cross-validation training. For each of these replicates, six-fold cross-validation training was performed to select the best hyperparameters for each replicate based on the Pearson correlation coefficient. For each replicate, therefore, 2,860 complexes were used for training, 572 for cross-validation, and 493 for testing.

The input features were robustly centered and scaled by the median and interquartile range (IQR) of the training set for each train-test split. All models obtained by the hyperparameter search were evaluated on the corresponding PDBbind test set as well as on the external test sets. We report the mean and standard deviation of the performance metrics.

### 2.4. Evaluation Metrics

To assess model performance, the root-mean-squared error (*RMSE*), Pearson (*r*), and Spearman (ρ) correlation coefficients, and the coefficient of determination, *R*^2^, were computed using the sklearn.metrics and scipy.stats Python modules. Additionally, we report the $Q_{F3}^{2}$ metric (Equation 2) (Consonni et al., 2009), as it is considered to be better suited for QSAR-like tasks than *R*^2^ (Todeschini et al., 2016).

(2) $$\begin{array}{r} Q_{F3}^{2}=1-\frac{\sum_{i}^{n_{test}}{\left(\hat{y}-y_{test}\right)}^{2}}{\sum_{i}^{n_{test}}{\left(\hat{y}-\bar{y_{train}}\right)}^{2}} \end{array}$$

### 2.5. Models and Hyperparameters

As part of this work, we evaluated different machine learning models. We considered linear regression (LR), as it was also used in the previous RASPD approach (Mukherjee and Jayaram, 2013), support vector regression (Drucker et al., 1997) (SVR), k-Nearest Neighbors (kNN), simple deep neural networks (DNN), random forests (Breiman, 2001) (RF), and a variant of the former, extremely random forests (Geurts et al., 2006) (eRF). The associated hyperparameters for each method were optimized by a grid search covering a typical space. Further details on each method and their associated hyperparameters are given in the Supplementary Materials. A comprehensive list of tested hyperparameters is given in Supplementary Table 2. All models except the neural networks were built using the scikit-learn Python package (version 0.20.2) (Pedregosa et al., 2011). For the neural networks, the Keras API (version 2.2.4) (Chollet et al., 2015) for TensorFlow (version 1.12) was used in conjunction with the talos package (version 0.4.6) (Kotila, 2018) for hyperparameter optimization.

### 2.6. Estimation of Feature Importance

To estimate the importance of individual input features, a simple permutation-based approach was used (Breiman, 2001). After prediction on a real-world test set, the column of each feature in the data set was shuffled in five replicates, and the mean change in Pearson correlation coefficient was computed. The model then has to make a prediction based on a random sample from a distribution with the same mean and variance. A drop in predictive performance indicates that the prediction is dependent on this feature.

### 2.7. Enrichment Analysis With Decoy Compounds From the DUD-E Dataset

To evaluate the performance of RASPD+ for capturing active molecules from a pool of computationally generated decoys, 3D coordinates of active and decoy molecules were retrieved from the DUD-E data set (Mysinger et al., 2012). This set contains 102 targets with on average ≈200 distinct and validated binding ligands and corresponding ≈14, 000 selected decoys for each system. Information about the proteins, as well as the number of active and decoy molecules for each system, is given in Supplementary Table 9. Enrichment was performed by selecting a given percentage of molecules that scored highest in the given method. For scoring, the predictions across the six cross-validation folds of a replicate were averaged. The enrichment factor was defined as the ratio of the fraction of active molecules in the enriched set divided by the fraction of the active molecules in the total set. For failure case analysis, we additionally determined which systems contained another cofactor in the binding pocket by checking for non-protein atoms within the pocket structure. Surface-only binding sites were identified by filtering interactions with few amino-acids and manually validating surface binding. More detailed subsets of DUD-E were adopted from Vieira and Sousa (2019) instead of the more coarse-grained classification from Mysinger et al. (2012).

## 3. Results

### 3.1. Analysis of the Descriptors and Data Sets

**Figure 2:**
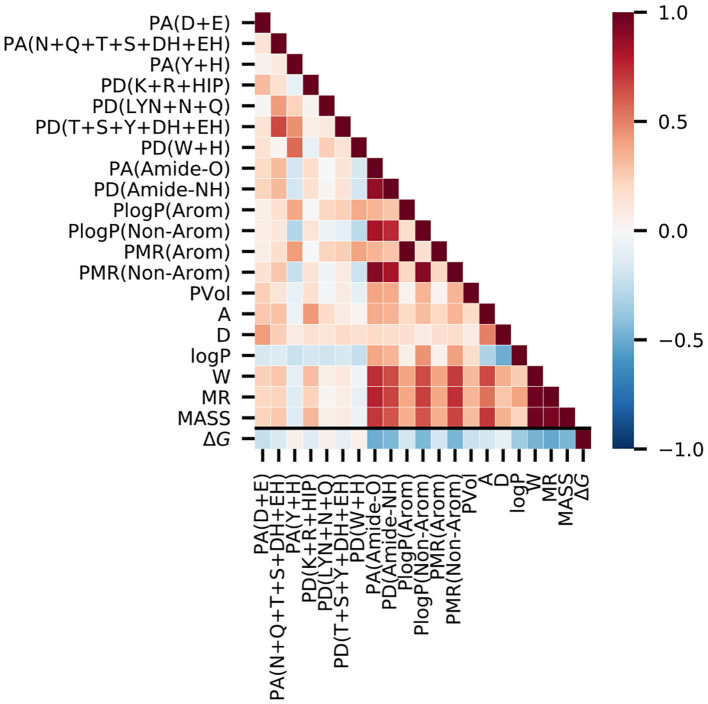
Correlation analysis on the PDBbind data set reveals that the experimental binding-free energy has the strongest negative correlation with the ligand molar refractivity (MR, Spearman ρ = −0.51), and with the number of peptide bond oxygen atoms (hydrogen bond acceptors) present in the putative protein binding pocket [PA(Amide-O), Spearman ρ = −0.49]. The value of the Spearman's correlation coefficient is indicated by color.

To confirm the usefulness of the chosen molecular descriptors, we performed correlation analysis on the PDBbind refined set (Figure 2, Supplementary Table 3). The Spearman correlations with the binding free energy, Δ*G*, were negative for most descriptors, as stronger binding is indicated by negative values of Δ*G*. The strongest negative correlations were observed for the molar refractivity of the ligand molecule (MR, −0.51) and the abundance of peptide bond oxygen atoms (hydrogen bond acceptors) inside the protein binding pocket [PA(Amide-O), −0.49]. The correlations with Δ*G* for the features for specific amino acids were lower than 0.25 in magnitude, which is less than the corresponding correlation (>0.4) obtained for the backbone [PA(Amide-O), PD(Amide-NH)] and non-aromatic amino acid [PlogP(Non-Arom), PMR(Non-Arom)] descriptors.

We next analyzed the correlations among the descriptors in particular to check for possible biases for certain interactions in the protein-ligand complexes of the PDBbind data. Amongst the ligand descriptors, the strongest correlations were observed between molecular weight, molar refractivity, and Wiener index (MASS, MR, and W). For the protein features, the strongest correlation was between the two descriptors for the aromatic amino acids, PlogP(Arom) and PMR(Arom). In addition, the backbone-based features, [PA(Amide-O) and PD(Amide-NH)], had a high correlation with the log P and molar refractivity values of the non-aromatic residues. Among the hydrogen bond contributions of the amino acids, we observed the strongest correlation with ρ = 0.66 between PD(T+S+Y+DH+EH) and PA(N+Q+T+S+DH+EH). This correlation is expected because they share the highest number of amino acids.

A higher correlation between the ligand and the protein features was observed between ligand features that directly scale with the size of the molecule (MASS, W, and MR) and the more general protein features, such as the backbone features and the log P and MR values of the non-aromatic residues. These protein features are expected to be related to the ligand size and, therefore, do not indicate any data set-specific bias of the PDBbind data set.

Comparing the distributions of binding-free energies Δ*G* between the PDBbind data set used for training and validation and the CSAR 2012 and 2014 (Dunbar et al., 2013; Carlson et al., 2016) and Wang et al. (2015) external data sets used for testing revealed that the PDBbind data set covers a wider range of binding free energies (Supplementary Figure 1). In contrast, the 101 protein-ligand complexes from the CSAR NRC-HiQ release cover a wider Δ*G* range than PDBbind.

From the distribution of the individual descriptors, it is clear that the PDBbind data set encompasses the full range of descriptor values covered by the other data sets (Supplementary Figure 2), even though there are differences in the mean values of the descriptors. For example, the average ligand molecular weight was lowest for the CSAR-NRC HiQ data and highest for the D3R data from CSAR 2012 and CSAR 2014.

### 3.2. Trained Models Random Forests Outperform Neural Networks

**Figure 3:**
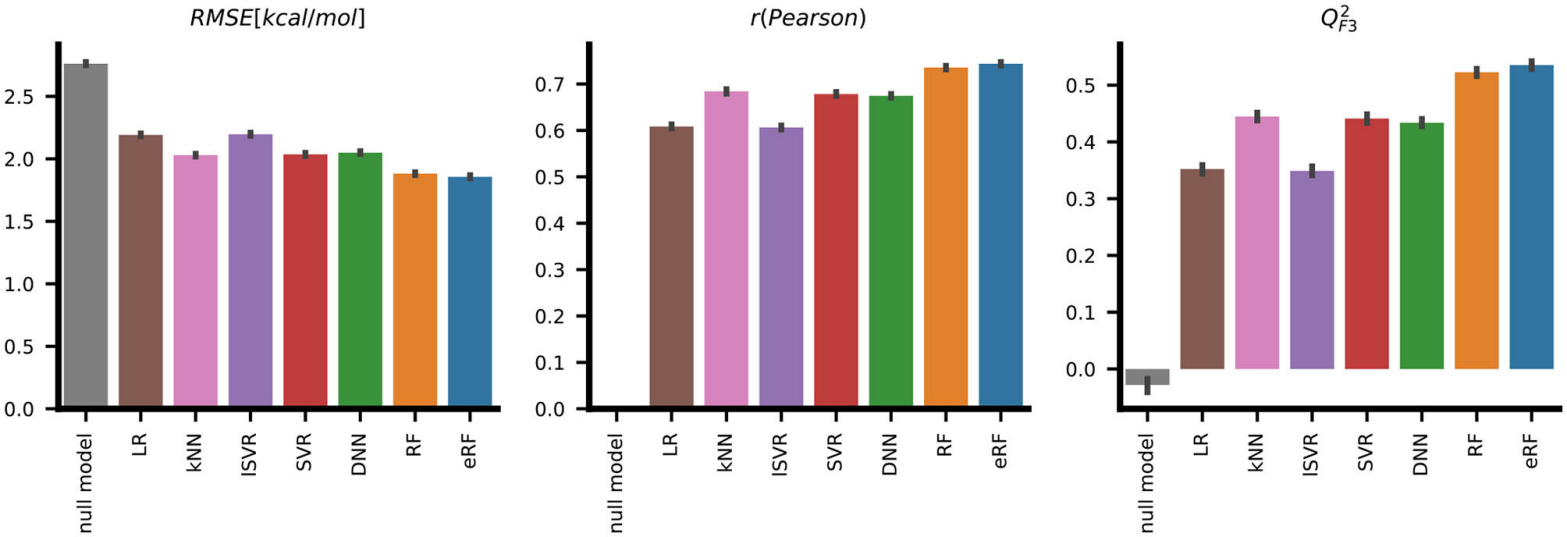
Systematic evaluation of the predictive performance of the seven different machine learning methods on the PDBbind test set shows that, according to three metrics, the extremely random forests (eRF) model performs better than models derived by the other machine learning methods in predicting protein-ligand binding free energy. The error bars indicate the standard deviation for 10 replicates with six-fold cross-validation.

Initial tests revealed high variability in the performance metrics that depended on a random training and validation data split. We thus chose a nested cross-validation strategy to find the machine learning models best suited for the chosen descriptors (Figure 1B). Therefore, performance metrics are reported as the mean of sixty models resulting from 10 random data set draws and six-fold cross-validation. The corresponding standard deviation enables the quantification of the uncertainty of the performance metrics. Apart from the baseline correlation values between the individual descriptors and the target variable Δ*G*, we included a null model, which simply predicted the mean Δ*G* of the training data, to verify predictive power. The root-mean-squared error, *RMSE*, of 2.76±0.05 measured for this null model is identical to the population standard deviation for the respective training folds (Table 1, Figure 3). The linear regression model derived by ordinary least squares fitting, similar to the original RASPD approach (Mukherjee and Jayaram, 2013), achieved a *RMSE* of 2.19±0.05 kcal/mol on the test set. We tested six other methods and assessed whether they improved on this value.

**Table 1:** Comparison of the performance of the models derived with seven different machine learning methods for predicting the protein-ligand binding free energy for the PDBbind test set.

**Model**	**RMSE**	**r**	**ρ**	**R^2^**	**$Q_{F3}^{2}$**
Null model	2.76 ± 0.05	0.0 ± 0.0	NA	−0.00 ± 0.00	−0.03 ± 0.05
LR	2.19 ± 0.05	0.61 ± 0.02	0.60 ± 0.02	0.37 ± 0.02	0.35 ± 0.03
kNN	2.03 ± 0.04	0.68 ± 0.02	0.67 ± 0.02	0.46 ± 0.03	0.44 ± 0.03
lSVR	2.20 ± 0.05	0.61 ± 0.02	0.60 ± 0.02	0.37 ± 0.02	0.35 ± 0.03
SVR	2.04 ± 0.05	0.68 ± 0.02	0.67 ± 0.02	0.45 ± 0.03	0.44 ± 0.03
DNN	2.05 ± 0.05	0.67 ± 0.02	0.66 ± 0.02	0.45 ± 0.02	0.43 ± 0.03
RF	1.88 ± 0.04	**0.74** **±0.02**	0.73 ± 0.02	0.53 ± 0.02	0.52 ± 0.02
eRF	**1.86** **±0.05**	**0.74** **±0.02**	**0.74** **±0.01**	**0.55** **±0.02**	**0.54** **±0.03**

*The five metrics of performance are given as mean and standard deviation values computed by averaging from the 10 different random test set splits and six cross-validation folds. The RMSE is given in kcal/mol. NA, not applicable*.

SVR with a Gaussian radial basis function (RBF) kernel and a neural network with two hidden layers performed with *RMSE* values of 2.04±0.05kcal/mol and 2.05±0.05 kcal/mol, respectively, these were similar to k-nearest neighbors with an *RMSE* of 2.03±0.04 kcal/mol. Superior performance in terms of both deviation, quantified by *RMSE*, and ranking, as measured by the Spearman correlation ρ, was achieved with the two random forest-based models. The eRF model had a *RMSE* of 1.86±0.05kcal/mol and a Pearson correlation *r* of 0.74±0.02, and the RF model performed similarly (Table 1, Figure 3).

**Figure 4:**
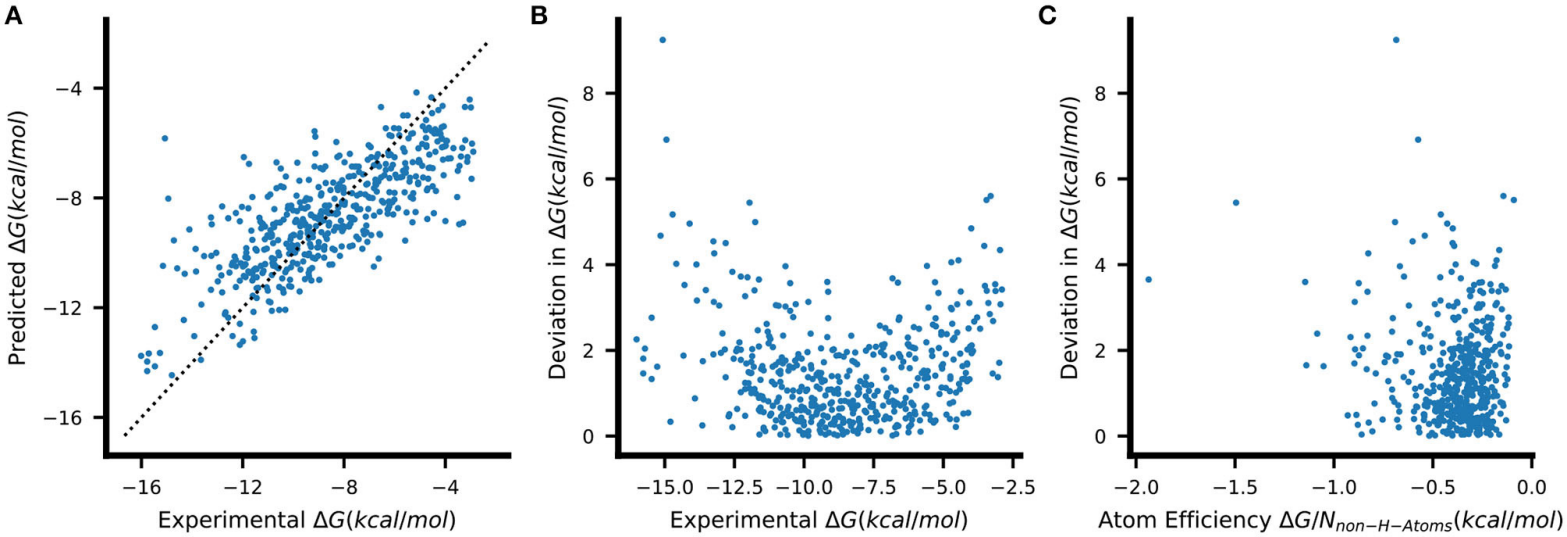
**(A)** Binding-free energies predicted by a single eRF model on unseen PDBbind test data. The dashed line indicates the ideal prediction. The absolute errors of the predictions shown in **(A)** are plotted against **(B)** the respective true Δ*G* and **(C)** the atom efficiency, which describes the Δ*G* contributed on average by each non-hydrogen atom (atom efficiency = Δ*G*/*N*_*non*−*H*−*atoms*_).

We therefore selected the resulting eRF models for further analysis. We note that these eRF regressors, which use 200 trees and have no limits on the number of samples per leaf, overfit the training set despite showing better validation set performance compared to more strongly regularized variants (see Supplementary Tables 4, 5). Nevertheless, an examination of the predictions of the eRF models on the PDBbind test data shows that the general trends in the data are captured although the lowest Δ*G* values are overestimated, and the highest Δ*G* values are underestimated (Figure 4A). The greatest deviations from the experimental values are thus observed for those complexes with extremely low or high binding free energies (Figure 4B). There is, however, no clear relation between having a higher error value and the atom efficiency (Figure 4C). The same trends were also observed with all the other machine learning methods.

### 3.3. Results on External Test Sets

To compare our RASPD+ approach using eRF models as well as LR with existing methods, we performed an evaluation on several external data sets from the literature (Dunbar et al., 2011, 2013; Wang et al., 2015; Carlson et al., 2016) that have different characteristics, as previously done by Jiménez et al. (2018) To compare to other methods for predicting protein-ligand binding free energy, we considered the previous RASPD approach (Mukherjee and Jayaram, 2013) as a method that does not rely on full docking, *K*_*DEEP*_ (Jiménez et al., 2018) as a representative deep learning-based method, RF-Score (Ballester and Mitchell, 2010) as a method using random forests, and cyScore (Cao and Li, 2014) and X-Score (Wang et al., 2002) as traditional docking scoring functions. Previously reported *RMSE* values (Jiménez et al., 2018) were transformed from errors in *pK* values to errors in Δ*G* for the comparisons. The RASPD+ eRF model consistently achieved lower error and higher correlation compared to the linear regression using the RASPD+ descriptors and this difference was more pronounced when comparing to the original RASPD linear regression model. With respect to the absolute deviation, measured by *RMSE*, the established scoring functions, RF-Score and X-Score performed best (Table 2). Only on set 2 of the challenging CSAR-NRC HiQ release (Dunbar et al., 2011) did RASPD+ with the eRF model have a lower *RMSE*, with a value of 2.23±0.04 kcal/mol, than the existing docking-based methods. When considering the Pearson correlation as a proxy for the ranking performance, RASPD+ with eRF models not only achieved the best result on the CSAR-NRC HiQ set 2 (*r* = 0.78±0.01) but also achieved *r* = 0.70±0.02 on the data set curated by Wang et al. (2015) (Table 2).

**Table 2:** Comparison of the performance of RASPD+ using eRF and LR models with five other methods to compute protein-ligand binding-free energy.

	**RASPD+**						
**Data set**	**eRF**	**LR**	**RASPD**	KDeep^*^	RF-Score^*^	CyScore^*^	X-Score^*^
**RMSE**							
CSAR HiQ 1	3.02 ± 0.04	3.07 ± 0.02	3.43	2.84	**2.71**	3.18	3.15
CSAR HiQ 2	**2.23** **±0.04**	2.44 ± 0.02	2.79	2.60	2.26	3.00	2.51
CSAR12	1.50 ± 0.02	1.68 ± 0.02	1.93	2.17	1.36	2.84	**1.27**
CSAR14	1.36 ± 0.03	1.64 ± 0.02	2.05	2.39	**1.19**	2.03	1.36
Wang et al.	1.39 ± 0.03	1.39 ± 0.02	2.00	1.47	**1.19**	5.74	1.49
**Pearson** **r**							
CSAR HiQ 1	0.62 ± 0.02	0.58 ± 0.01	0.54	0.72	**0.77**	0.65	0.60
CSAR HiQ 2	**0.78** **±0.01**	0.68 ± 0.01	0.67	0.65	0.75	0.64	0.65
CSAR12	0.40 ± 0.03	0.25 ± 0.01	0.29	0.37	0.46	0.26	**0.48**
CSAR14	0.55 ± 0.03	0.23 ± 0.02	0.32	0.61	0.80	0.67	**0.82**
Wang et al.	**0.70** **±0.02**	0.68 ± 0.01	0.55	0.29	0.24	0.27	0.25

*The RMSE [kcal/mol] and Pearson correlation coefficients for predictions on five external test sets are given. RASPD used simpler descriptors and the LR parameters from Mukherjee and Jayaram (2013). The values for the other methods are taken from Jiménez et al. (2018). The values for the best performing models are shown in bold*.

* *pK values reported by Jiménez et al. (2018) were converted to ΔG for comparison of RMSE values*.

The good performance of the RASPD+ eRF on the Wang et al. data set is also borne out in the distribution of predictions (Supplementary Figure 3), which, compared to the results on CSAR-NRC HiQ (Supplementary Figure 6), not only ranks but also faithfully captures the range of energies. On both the CSAR 2012 and CSAR 2014 data sets, clear failures of the RASPD+ eRF and most other methods can be observed. For some cases, the RASPD+ model predicts energies in a very narrow range around -10.5 kcal/mol (Supplementary Figures 4, 5), but, interestingly, this value does not correspond to the mean Δ*G* value for the training data.

As the CSAR 2012 (Dunbar et al., 2013) and CSAR 2014 (Carlson et al., 2016) releases and the data set from Wang et al. (2015) provided data for several ligands for each individual protein target, we analyzed the failure cases at the level of the individual proteins (Supplementary Tables 6–8). The Pearson and Spearman correlations are below 0.3 for the BACE and CDK2 systems from the Wang et al. set (Wang et al., 2015) and CHK1 and SYK in the CSAR sets. In contrast, the CDK2 complexes in the CSAR 2012 set (Dunbar et al., 2013) achieved a Pearson correlation of *r* = 0.50±0.05. The highest correlations were observed for the PTP1B, Mcl-1, TYK2 systems in the Wang et al. (2015) data (Supplementary Table 6) and for the CDK2-Cyclin A complex (Supplementary Table 7) and TrmD on the CSAR data (Supplementary Table 8). Strikingly, only for PTP1B, TYK2, and TrmD was *R*^2^ > 0.3 observed while all $Q_{F3}^{2}$ values were above 0.5.

### 3.4. Feature Importance Analysis

**Figure 5:**
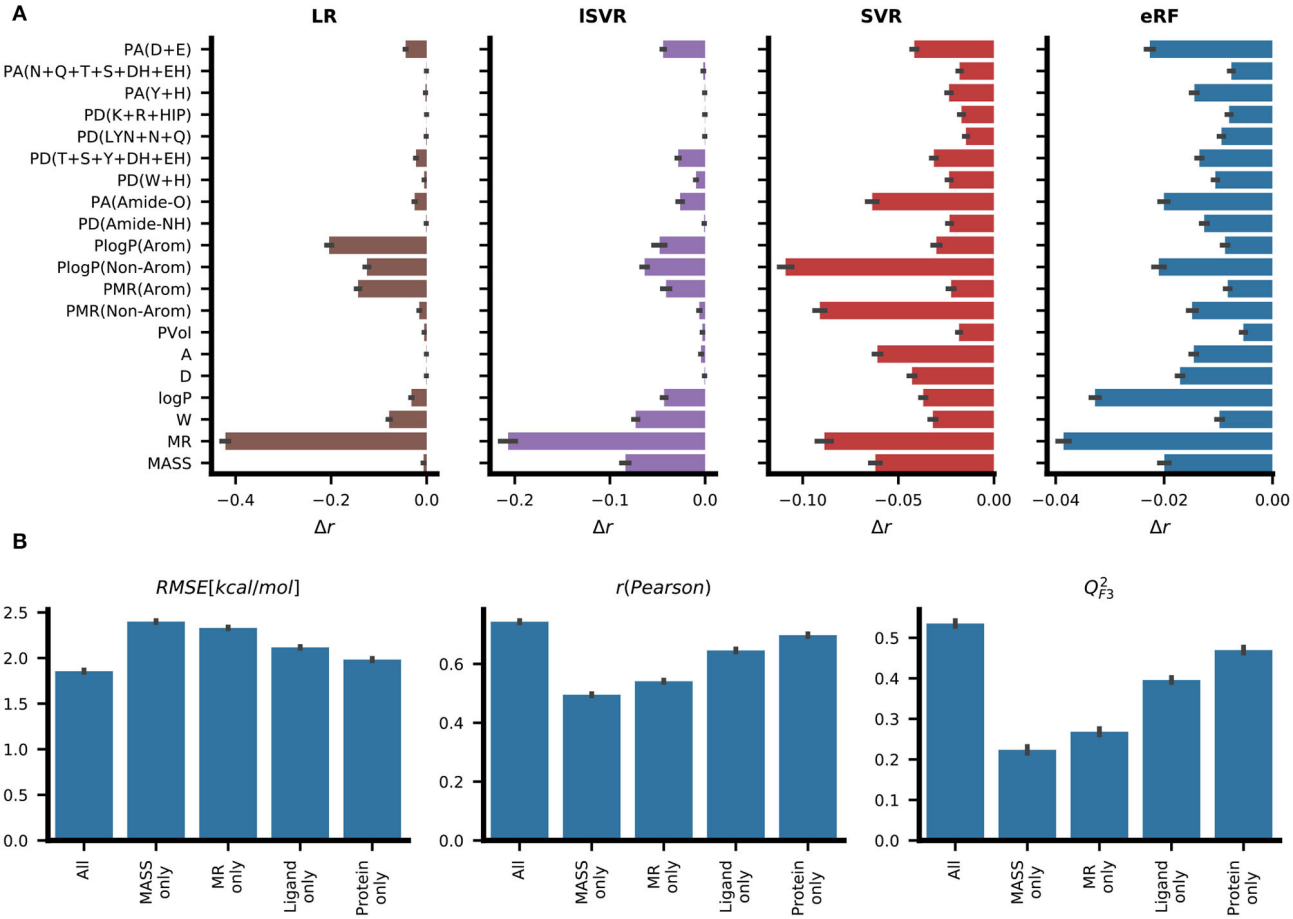
Analysis of feature importance for the predictive performance of RASPD+. **(A)** The average change in predictive performance as measured by Pearson correlation when the corresponding feature column was shuffled. Results are reported for linear regression (LR), linear support vector regression (lSVR), support vector regression (SVR), and extremely random forests (eRF). **(B)** Ablation based analysis entailing training eRF models with different feature sets. The models trained on just the values for molar refractivity (MR only) and molecular weight (MASS only) serve as lower bounds for measuring performance. The “ligand only” and “protein only” models trained on either only ligand or only protein features, respectively, perform better than the “MR only” and “MASS only” models but not as well as the models derived from all features. The protein features contain implicit information on the ligand size and this may indicate why the performance of the “protein only” model is better than that of the “ligand only” model (see Figure 1 for details).

To assess which features contribute to accurate predictions, two strategies were chosen. By permutation feature importance, the contribution to the prediction was quantified by the change in the Pearson correlation coefficient after shuffling the values in the individual feature columns randomly. Three different model types – namely, linear regression, support vector machine, and extremely random forests – showed different relative contributions of the individual features (Figure 5A). While LR assigned high contributions to a few features, the reduction in predictive performance for each shuffled feature was lower for eRF and the contribution signal was more evenly distributed among the different features. Molar refractivity (MR), which was the feature most strongly correlated with the target variable Δ*G*, showed the strongest effect in the LR, lSVR, and eRF models. For LR, randomizing MR almost completely removed the predictive power (*r* < 0.2). Among the protein features, the LR, SVR, and RF methods showed high contributions for the general descriptor PlogP(Non-Arom) (Figure 5A). While both SVR and eRF assigned high contributions to the PMR(Non-Arom), LR, and lSVR placed higher contributions on PlogP(Arom) and PMR(Arom) among the general protein features. PA(Amide-O), which had the second-highest correlation with Δ*G*, showed a pronounced signal for the editedSVR and eRF models. The hydrogen bond acceptor count at the negatively charged amino acid residues [PA(D+E)] was informative for all these machine learning methods. In the eRF model, it had an importance value similar to the general protein features, such as the residue log P values. This is especially surprising as no information on the ligand charge was provided, and the count of positively charged amino acid hydrogen bond donors [PD(K+R+HIP)] did not contribute strongly to the predictions.

Additionally, we trained eRF models on subsets of the features and compared their performance to the full model (Figure 5B). Among the models trained on a single feature, the model trained on molar refractivity (MR) achieved better performance than that trained on molecular weight (MASS). Models trained on just the features of the protein pocket performed better than models using only ligand descriptors. In this case, the protein features still contained information about the ligand implicitly, as each protein descriptor is dependent on the size of the sphere surrounding the ligand. These reduced feature set models were also subjected to permutation feature importance analysis. For models with only ligand features, a very similar ranking of ligand features compared to the full training set was observed, illustrating the general preference for using those features for prediction (Supplementary Figure 7).

When examining the feature importance for protein-only models, the backbone hydrogen bond acceptor [PA(Amide-O)] stands out compared to the feature importance on the full feature set (Supplementary Figure 8). This could be partially explained by the fact that this feature showed a strong correlation with general ligand features (Figure 2) and thereby provides information related to general ligand size.

### 3.5. Enrichment of Active Molecules From the DUD-E Data Set

To assess the usefulness of our RASPD+ method, we simulated a drug discovery setting using the benchmark DUD-E data set, which contains several computationally generated decoys per active compound (Mysinger et al., 2012). For each of the seven machine learning models, we calculated enrichment factors (EF) to quantify how effective ranking by predicted binding free energies was at enriching active molecules from the whole data set (Table 3). We also compared the RASPD+ results with those of RASPD (Mukherjee and Jayaram, 2013) and found that the linear regression models of both RASPD+ and RASPD were the most effective when filtering to 1, 5, and 10% of the samples, with EFs of 2.7±3.4 and 4.1±4.0, respectively, when filtering down to 1 % of the samples. The high standard deviation in the mean EF resulted from high variability in the performance of different methods on individual proteins (Supplementary Table 9). As methods that ranked on average less favorably provided the only acceptable enrichment on some of the systems, we chose a conservative approach to interpreting the results by combining the predictions of all the methods. We thus also considered the union of the sets of top candidate molecules from all seven machine learning models. This combination achieved an enrichment at 1 % of 2.4±2.3, similar to the linear methods. By excluding the predictions of the worst-performing method kNN (Union w/o kNN), this set improved to 2.5±2.7. When only combining the predictions of the three methods performing best on the DUD-E set LR, lSVR, and SVR (Union Top 3), this further increased to 2.8±3.6. For comparison, the performance of scoring functions functions based on the docked structures of ligand-protein complexes on the DUD-E set has been assessed by Chen et al. (2019). The highest early (1 %) and late (10 %) enrichment factors were 6.67 and 2.55, respectively, and were obtained using the knowledge-based DLIGAND2 scoring method, whereas the corresponding values obtained with the widely used AutoDock Vina scoring function were 5.12 and 2.60. The late EF obtained with the RASPD+ union w/o kNN approach is similar to that of these docking-based methods.

**Table 3:** Average enrichment factors with corresponding standard deviations for the top 1, 5, and 10% of the data selected from the DUD-E systems.

	**Full DUD-E**			**w/o cofactor/surface binding**		
	*******n*******_******systems******_ = 102			*******n*******_******systems******_ = **55**		
**Method**	**EF 1 %**	**EF 5 %**	**EF 10 %**	**EF 1 %**	**EF 5 %**	**EF 10 %**
eRF	1.8 ± 2.5	1.5 ± 1.4	1.3 ± 0.9	2.0 ± 3.0	1.7 ± 1.5	1.0 ± 1.0
RF	1.9 ± 2.4	1.5 ± 1.5	1.3 ± 1.0	2.3 ± 2.8	1.7 ± 1.7	1.4 ± 1.1
DNN	2.0 ± 2.0	1.4 ± 1.2	1.3 ± 0.9	1.8 ± 2.3	1.6 ± 1.3	1.4 ± 0.9
kNN	1.5 ± 1.9	1.3 ± 1.3	1.2 ± 0.9	1.8 ± 2.4	1.6 ± 1.6	1.0 ± 1.0
lSVR	2.6 ± 3.1	1.9 ± 1.6	1.6 ± 1.1	2.6 ± 3.3	2.0 ± 1.7	1.7 ± 1.2
SVR	2.3 ± 3.5	1.6 ± 1.5	1.4 ± 1.0	3.1 ± 4.3	1.9 ± 1.8	1.5 ± 1.1
LR	2.7 ± 3.4	2.0 ± 1.7	1.7 ± 1.2	2.9 ± 3.7	2.1 ± 1.8	1.8 ± 1.3
RASPD	4.1 ± 4.0	2.2 ± 1.7	1.7 ± 1.1	4.2 ± 4.2	2.3 ± 1.7	1.7 ± 1.2
Mean ensemble	2.0 ± 3.0	1.8 ± 1.6	1.6 ± 1.1	2.6 ± 3.4	2.0 ± 1.8	1.7 ± 1.1
Union	2.4 ± 2.3	1.8 ± 1.4	1.6 ± 1.0	2.8 ± 2.6	2.1 ± 1.6	1.8 ± 1.1
Union w/o kNN	2.5 ± 2.7	1.8 ± 1.5	2.0 ± 1.0	3.0 ± 3.0	2.1 ± 1.7	1.7 ± 1.1
Union Top 3	2.8 ± 3.6	1.9 ± 1.7	1.6 ± 1.2	3.3 ± 4.2	2.0 ± 2.0	1.8 ± 1.3

*Union is the enrichment achieved by selecting the non-redundant set of candidate compounds obtained by combining the selections of each method. Performance when excluding cofactor and surface binding sites is also reported. Values are also given for the original RASPD method*.

One of the reasons for some of the poor predictions with DUD-E is that, in contrast to the training with PDBbind, the query ligands may bind at a different position to the co-crystallized ligand in the target whose center of mass is used to define the binding site for which protein properties are computed. If the query ligand binds in a somewhat different position, the computed protein features may not be so relevant. From the feature importance analysis (Figure 5), we see that for the eRF model, all the features contribute in a similar way to the final prediction. In contrast, for the LR and lSVR models, the dominant contributions to the prediction were from ligand molar refractivity and just three out of the 15 protein features. Since, in the LR and lSVR models, only a few protein features contribute to the final score, erroneous protein features may have less impact on the final predicted value compared to the random forest-based models. The average EF values for the LR method, and for the original RASPD LR model, for all the DUD-E sets are therefore higher than for the other methods. Another reason for low EF values for some targets is the presence of cofactors or structural water molecules in the binding site in some proteins as well as highly solvent exposed binding sites. To assess how much the performance is impacted by situations not properly modeled by RASPD+, we also considered whether a cofactor in the binding site or a mostly solvent-exposed surface binding site affects performance. For most methods, the exclusion of those challenging pockets, which by design could not be fully modeled with RASPD and RASPD+ descriptors, improved the mean performance (Table 3).

Additionally, we analyzed the performance of the different protein subgroups in the DUD-E set (Table 4). Here we observed the lowest average performance for the protease subgroup when considering the union over all methods and when considering only the top three union, cytochrome P450, and nuclear receptor targets were the groups with the lowest enrichment. The poor performance for cytochrome P450s may be due to the heme cofactor in their binding site whereas it is notable that the eight proteases (out of the 11 in the DUD-E data set) with low EF factors have ligands that are solvent exposed in the crystal structure or there are structural water molecules bridging between polar atoms of the ligand and the protein. The highest enrichment was observed for ion channels, G-protein coupled receptors (GPCR), and kinases in both settings (Table 4).

**Table 4:** Mean enrichment factors for different subsets of the DUD-E set.

		**Union**			**Union top 3**		
**Target subset**	**Count**	**EF 1 %**	**EF 5 %**	**EF 10 %**	**EF 1 %**	**EF 5 %**	**EF 10 %**
Cytochrome P450	2	1.86	1.25	1.28	1.11	1.65	1.42
GPCR	5	4.30	2.94	2.18	7.37	3.61	2.61
Ion channel	2	5.54	2.15	1.61	7.67	2.16	1.56
Kinase	23	3.04	2.55	2.15	4.43	3.03	2.49
Metal containing enzyme	18	2.47	1.66	1.42	2.89	1.84	1.55
Miscellaneous	30	1.83	1.49	1.41	1.57	1.34	1.12
Nuclear receptor	11	1.73	1.18	1.11	1.14	0.93	1.01
Protease	11	1.56	1.62	1.41	1.91	1.75	1.60

*Performance reported both the union over the predictions of all methods and the union of the predictions from the three best ranking methods (LR, lSVR, and SVR)*.

## 4. Discussion

As the global health crisis surrounding the SARS-CoV-2 pandemic (Wu et al., 2020) has demonstrated, there is a need for fast computational tools to accelerate drug design and development processes. The method we present here, RASPD+, is able to perform virtual screening of large libraries of compounds (Irwin and Shoichet, 2005; Wishart et al., 2006) at a fraction of the time typically required for protein-ligand docking methods. This enables quick prioritization of candidates for a follow up with more accurate yet computationally more demanding methods, such as docking. We achieved the speed up by training machine learning models on simple pose-invariant ligand and protein descriptors. With this simplified approach, we achieved results comparable to existing scoring functions (Wang et al., 2002; Ballester and Mitchell, 2010; Cao and Li, 2014; Jiménez et al., 2018) when predicting the binding free energy, Δ*G*, on several data sets. By splitting the PDBbind training, testing, and validation data in a nested cross-validation setup, we were able to assess reliably that random forest models, particularly the extremely random forest model, performed best on this type of data. While this splitting strategy increases confidence in the comparison of learning methods and feature importance analysis within the study, other data set splitting strategies, which explicitly control how similar proteins or ligands are between training and test sets (Feinberg et al., 2018; Sieg et al., 2019; Su et al., 2020), may be more appropriate to assess performance on completely different ligands or proteins directly.

We accounted for this deficiency by not only testing the regression performance on different external test sets but also by assessing the ability of the RASPD+ models to enrich active molecules from a set of inactive decoys. Although the achievable enrichment factors were not as high as state-of-the-art docking or free energy prediction methods (Li et al., 2014), RASPD+ still displayed appreciable enrichment of active molecules on the DUD-E data set (Mysinger et al., 2012). RASPD+ was able-without sampling docking poses-to achieve similar performance to an older scoring function in a docking method comparison (Li et al., 2013; Chen et al., 2019). This is remarkable for two reasons: First, the training set only includes molecules displaying binding to their specific target protein. Secondly, four of the six physicochemical descriptors (molecular weight, hydrogen bond donor and acceptor count, and logP value), used to describe the ligand molecule, were initially used to select decoys similar to the active molecules for the DUD-E data set (Huang et al., 2006; Mysinger et al., 2012). This makes the task of distinguishing active and inactive molecules particularly difficult for our models that employ only basic ligand descriptors (Lagarde et al., 2015). Notably, however, molar refractivity (MR), which was not used for the creation of the DUD-E decoys, was not only a powerful predictor on its own (*r*>0.5) but was also consistently assigned the highest feature importance among the ligand features. The high importance of MR agrees with results from a recent study that used ligand descriptors to enhance the performance of a common docking scoring function (Boyles et al., 2020).

Not considering pockets containing metal ions or other cofactors, which are not taken into account by the simple RASPD+ descriptors, yielded slightly higher average enrichment than on the full DUD-E set. Random forest methods, which were best suited for the Δ*G* regression on known binders, were for most proteins outperformed by the simpler linear regression methods. This observation might support the recent finding that random forest methods, in particular, benefit from highly similar training molecules (Su et al., 2020). Considering the strengths and weaknesses of the different machine learning methods, we therefore recommend that for applications of RASPD+, the results of the seven different machine learning methods are combined by picking top candidates from the rankings produced by each method. For this, we demonstrated different combinations using the union of selection sets from the different methods.

If this approach is applied to pick the top 10 % of RASPD+ candidates, this can provide a 10-fold reduction in the time spent for docking. Notably, we achieved computation times for RASPD+ that were over 100 times faster than Glide SP docking (Friesner et al., 2004) (Schrödinger Release 2019-4: Glide, Schrödinger, LLC, New York, NY) on a laptop grade CPU (data not shown), meaning that computation times for RASPD+ screening are negligible compared to times for docking and molecular dynamics simulation.

Thus, the use of RASPD+ is clearly beneficial in time-critical applications of virtual screening of large compound libraries against individual protein targets. Moreover, higher structure-based screening throughput could also enable more effective inverse virtual screening of protein databases to assess the specificity and potential side-effects of candidate molecules.

## Data Availability Statement

All data generated in this work necessary to reproduce the findings are available at https://doi.org/10.5281/zenodo.3937425 and the RASPD+ software is available on GitHub at https://github.com/HITS-MCM/RASPDplus.

## Author Contributions

GM developed the descriptor pipeline and performed enrichment analysis. LA and SH trained and evaluated machine learning models. SH performed feature importance analysis. SH, LA, GM, and RW analyzed the data. BJ provided advice for the development of the descriptor pipeline. RW provided guidance and supervised the work with GM. SH wrote the manuscript with input from all authors.

## Conflict of Interest

The authors declare that the research was conducted in the absence of any commercial or financial relationships that could be construed as a potential conflict of interest.

## Supplementary Material

The code used for creating the models and figures in this paper, and scripts to run the RASPD+ pipeline are available on GitHub: https://github.com/HITS-MCM/RASPDplus. Precomputed descriptor values and the model weights can be downloaded from https://doi.org/10.5281/zenodo.3937425.
