# Supplementary material for: RASPD+: Fast Protein-Ligand Binding Free Energy Prediction Using Simplified Physicochemical Features
Source: Front Mol Biosci. 2020 Dec 17;7:601065. doi: 10.3389/fmolb.2020.601065 (PMC7773945; doi:10.3389/fmolb.2020.601065)
Supplement: Supplementary file 1 [file Data_Sheet_1.PDF]

Supporting Information: RASPD+: Fast protein-ligand binding free energy prediction using simplified physicochemical features

Stefan Holderbach,[†,‡] Lukas Adam,[†,‡] B. Jayaram,[¶] Rebecca C. Wade,[*,†,§,‖] and Goutam Mukherjee[*,†,§]

†*Molecular and Cellular Modelling Group, Heidelberg Institute of Theoretical Studies, Schloss-Wolfsbrunnenweg 35, 69118 Heidelberg, Germany*

‡*Institute of Pharmacy and Molecular Biotechnology (IPMB), Heidelberg University, Im Neuenheimer Feld 364, 69120 Heidelberg, Germany*

¶*Supercomputing Facility for Bioinformatics & Computational Biology, Department of Chemistry, Kusuma School of Biological Sciences, Indian Institute of Technology Delhi, Hauz Khas, New Delhi, 110016, India*

§*Center for Molecular Biology (ZMBH), DKFZ-ZMBH Alliance, Heidelberg University, Im Neuenheimer Feld 282, 69120 Heidelberg, Germany*

‖*Interdisciplinary Center for Scientific Computing (IWR), Heidelberg University, Im Neuenheimer Feld 205, 69120 Heidelberg, Germany*

E-mail: rebecca.wade@h-its.org; goutam.mukherjee@h-its.org

List of Tables

SI 1 Content and origin of the datasets of protein-ligand complexes from D3R (`drugdesigndata.org`)[1] - CSAR12 and CSAR14 - and from Wang et al.[2] . . 9

SI 2 Hyperparameters evaluated for the different machine learning methodologies. The combinatorial space was explored by a naive grid search over all reasonable combinations. The most frequently chosen hyperparameter configuration is indicated in bold font. 10

SI 3 Values of the Spearman correlation between the protein and ligand descriptors and ΔG in the PDBbind refined data set[3]. The highest correlation is observed for MR and shown in bold. 11

SI 4 Comparison of the performance of different models on the PDBbind training set using five different metrics. Results for the different random test set splits and cross-validation folds were averaged and the mean and standard deviation are reported. The RMSE is given in kcal/mol. NA: Not applicable 12

SI 5 Comparison of the performance of different models on the PDBbind validation set using five different metrics. Results for the different random test set splits and cross-validation folds were averaged and the mean and standard deviation are reported. The RMSE is given in kcal/mol. NA: Not applicable 12

SI 6 Performance of the model for the individual protein targets provided by Wang et al.[2] 13

SI 7 Performance of the model for the individual protein targets in the CSAR 2012 data set.[4] 13

SI 8 Performance of the model for the individual protein targets in the CSAR 2014 data set.[5] 13

SI 9 Enrichment factors for the 102 targets of the DUD-E data set for different fractions of the total data selected.[6] Union describes the combination of all machine learning methods, where redundant molecules are removed. 14

List of Figures

SI 1 Distribution of binding free energy (ΔG) in the protein-ligand complexes in the training and external data sets. D3R contains protein-ligand complexes from both the CSAR 2012 and 2014 releases.[4,5] 27

SI 2 Distribution of the protein and ligand descriptor values in the training set and the external test sets shown colored by data set. The vertical lines indicate the mean values for the respective data sets. D3R contains protein-ligand complexes from both the CSAR 2012 and 2014 releases.[4,5] 28

SI 3 Individual predictions on the external data set from Wang et al.[2] (A) Predicted ΔG against experimental ΔG. (B) Error for each prediction against the experimental ΔG. (C) Error for each prediction against the atom efficiency ($\Delta G/N_{non-H-atoms}$). 29

SI 4 Individual predictions on the external CSAR 2012 data set. (A) Predicted ΔG against experimental ΔG. (B) Error for each prediction against the experimental ΔG. (C) Error for each prediction against the atom efficiency ($\Delta G/N_{non-H-atoms}$). 29

SI 5 Individual predictions on the external CSAR 2014 data set. (A) Predicted ΔG against experimental ΔG. (B) Error for each prediction against the experimental ΔG. (C) Error for each prediction against the atom efficiency ($\Delta G/N_{non-H-atoms}$). 30

SI 6 Individual predictions on the external CSAR-NSR HiQ data set. (A) Predicted ΔG against experimental ΔG. (B) Error for each prediction against the experimental ΔG. (C) Error for each prediction against the atom efficiency ($\Delta G/N_{non-H-atoms}$). 30

SI 7 Feature importance for models trained with only the ligand descriptors. The feature importance is computed as the average change in Pearson correlation coefficient for five permutations of the respective feature column by shuffling the data (see Methods for details.) 31

SI 8 Feature importance for models trained with only the protein descriptors. Feature importance is computed as the average change in Pearson correlation coefficient for five permutations of the respective feature column by shuffling the data (see Methods for details). 31

Supplementary methods

Details regarding the different machine learning methods used in this study.

Linear Regression (LR)

In the previous RASPD approach[7], the simplifying assumption was made that the dependent variable y, binding free energy, follows the physicochemical features x with a linear relationship. Therefore, all contributions behave purely additively. The parameters w and b in such a linear regression model (eqn. 1) can be fit to the data with the ordinary least squares algorithm.

$$\hat{y} = w^T \cdot x + b = \sum_{i}^{n_{features}} w_i \cdot x_i + b \tag{1}$$

Thus, if the features are linearly independent and possess a linear relationship with the output value, the coefficients w are informative about the relationship of the features to the predicted value in the model.

k-Nearest Neighbor (kNN)

A naive way to capture nonlinear relationships between the input features x and the output value y is to rely on a nearest-neighbor based approach to assign the binding free energy values of the closest training examples in feature space. As Euclidean distance is most commonly used, no explicit prioritization of features is enforced but dependent on the separation of training data points in the feature space. A tunable hyperparameter controls how many of the k closest neighbors are averaged to obtain a prediction $\hat{y}$.

Support Vector Regression (SVR)

Another approach to achieve separation based on distance in the feature space is *support vector regression*[8]. Here, a regression function is described based on the inner product of

the input features and a set of the training examples, the support vectors. Regularization is applied to minimize the contribution of support vectors within an error margin ϵ allowing to tune the bias-variance trade-off with an additional regularization parameter C. The necessary inner product can be computed not only linearly on the feature space but also in a high dimensional space using a kernel function as a similarity measure, thus allowing for nonlinear models. The most common kernel is the *radial basis function (rbf)* which decays with distance in feature space according to a Gaussian density function.

Neural Networks – Deep Learning (DNN)

Neural networks introduce nonlinearity and the ability to capture complex relationships by stacking multiple layers of linear matrix multiplication operations interleaved with a nonlinear function f.

$$\begin{aligned} h_0 &= x \\ \textbf{for } & n \text{ layers} \\ h_i &= f(w_i h_{i-1} + b_i) \\ y &= h_n \end{aligned} \tag{2}$$

Such a nonlinear activation function f could simply be a *Rectified Linear Unit (ReLU)*[9] or an *Exponential Linear Unit (ELU)*[10], which has the additional benefit of being continuous and smooth.

$$ReLU(x) = \max(0, x) \tag{3}$$

$$ELU(x) = \begin{cases} x & \text{if } x \geq 0 \\ e^x - 1 & \text{otherwise} \end{cases} \tag{4}$$

The number and size of these layers have to be chosen according to the complexity of the problem. To avoid overfitting, dropout regularization[11] with an additional dropout probability hyperparameter can be adopted. Training of the neural network models was performed using mini-batch stochastic gradient descent[12] with mean squared error as the loss function. This leads to the additional training hyperparameters of batch size, learning rate, and the number of epochs (full iterations over the training set).

Random Forests – Decision Tree Ensembles

Decision trees try to separate data by recursively finding features and thresholds that split the data into two groups with the most dissimilar output value, which are on the other hand as similar as possible within each group. As single decision trees tend to overfit the training data, several weak decision tree regressors are combined in their predictions by bagging resulting in *random forests* (RF)[13]. To generate independent regressors based on the same training data, two strategies are used: In the original random forest implementation, bootstrap samples of the training data for each tree increase diversity[13]. Another approach termed *extremely random forests* (eRF) or *extra trees* sets the decision boundary for a given feature at random[14]. Thus, the algorithm only picks the best-separating feature given the random boundary rather than additionally computing the best boundary. In both methods, only a random subset of the available features is used at each branch point when searching for the best-separating feature. To control the bias-variance trade-off, several hyperparameters can be set: The number of trees controls the achievable bias and regularizes the variance until the number of trees exceeds the number of truly independent tree samples. The method by which subsets of features are chosen determines how diverse or random the resulting trees are. Additionally, the number of splits can be controlled by a limit to how many samples can be placed in a leaf node.

Supplementary tables

Table SI 1: Content and origin of the datasets of protein-ligand complexes from D3R (`drugdesigndata.org`)[1] - CSAR12 and CSAR14 - and from Wang et al.[2]

Data set	Protein target	PDB ID	# ligands	Source of ligand structures and experimental affinities
CSAR12	Urokinase	5YC6	35	https://drugdesigndata.org/php/file-download.php?type=extended&id=76
	CDK2-Kinase	1H1Q	25	https://drugdesigndata.org/php/file-download.php?type=extended&id=99
	CDK2-CyclinA	4GCJ	23	https://drugdesigndata.org/php/file-download.php?type=extended&id=111
	CHK1-Kinase	2YEX	110	https://drugdesigndata.org/php/file-download.php?type=extended&id=70
	ERK2	4ZZN	298	https://drugdesigndata.org/php/file-download.php?type=extended&id=71
	LpXc	3UHM	20	https://drugdesigndata.org/php/file-download.php?type=extended&id=73
CSAR14	SYK	5LMA	583	https://drugdesigndata.org/php/file-download.php?type=extended&id=74
	tRMD	4YQD	31	https://drugdesigndata.org/php/file-download.php?type=extended&id=75
	HSP90	4YKU	146	https://drugdesigndata.org/php/file-download.php?type=extended&id=100
Wang et al.	BACE	4DJW	36	Ligand 2D-structure:[15]; experimental parameters: SI of[2]
	CDK2	1H1Q	16	Ligand 2D-structure:[16]; experimental parameters: SI of[2]
	MCL1	4HW3	67	Ligand 2D-structure:[17]; experimental parameters: SI of[2]
	p38	3FLY	34	Ligand 2D-structure:[18]; experimental parameters: SI of[2]
	PTP1B	2QBS	48	Ligand 2D-structure:[19]; experimental parameters: SI of[19]
	Thrombin	2ZFF	11	Ligand 2D-structure:[20]; experimental parameters: SI of[20]
	TYK2	4GIH	71	Ligand 2D-structure:[21,22]; experimental parameters: SI of[2]

Table SI 2: Hyperparameters evaluated for the different machine learning methodologies. The combinatorial space was explored by a naive grid search over all reasonable combinations. The most frequently chosen hyperparameter configuration is indicated in bold font.

Model	Parameter	Values
(l)SVR	C	0.01, **0.1**, 1, **10**
	ϵ	0, **0.1**
	Kernel	linear, gaussian rbf
	kernel parameter γ	`scikit-learn auto`
kNN	k	1, **5**, 10, 20
	distance metric	**Euclidean(**$L2$**)**
RF	Number of trees	20, 100, **200**
	Ratio of features	log_2, *sqrt*
	min. number of samples per leaf	**1**, 3, 5
eRF	Number of trees	20, 100, **200**
	Ratio of features	log_2, *sqrt*
	min. number of samples per leaf	**1**, 3, 5
DNN	Number of hidden layers	**2**, 3
	Activation Function	ReLU, **ELU**
	Dropout Probability	0, **0.1**, 0.2
	Hidden layer size	10, 20, [**layer 1: 20, layer 2: 10**]
	Optimizer	**SGD**, Adam
	Learning rate	**0.01**, 0.003, 0.001
	Loss function	**MSE**
	Initializer	variance scaling ($\sqrt{a/\text{fan-in}}$) $a = \mathbf{1}, 2$, **normal**/uniform

Table SI 3: Values of the Spearman correlation between the protein and ligand descriptors and ΔG in the PDBbind refined data set[3]. The highest correlation is observed for MR and shown in bold.

Descriptor	$\rho(\Delta G, Descriptor)$
PA(D+E)	-0.240
PA(N+Q+T+S+DH+EH)	-0.150
PA(Y+H)	0.061
PD(K+R+HIP)	-0.120
PD(LYN+N+Q)	0.060
PD(T+S+Y+DH+EH)	-0.087
PD(W+H)	0.053
PA(Amide-O)	-0.490
PD(Amide-NH)	-0.447
PlogP(Arom)	-0.190
PlogP(Non-Arom)	-0.450
PMR(Arom)	-0.187
PMR(Non-Arom)	-0.460
PVol	-0.224
A	-0.187
D	-0.091
logP	-0.364
W	-0.474
MR	**-0.508**
MASS	-0.454

Table SI 4: Comparison of the performance of different models on the PDBbind training set using five different metrics. Results for the different random test set splits and cross-validation folds were averaged and the mean and standard deviation are reported. The RMSE is given in kcal/mol. NA: Not applicable

Model	RMSE	r	ρ	R^2	Q^2_{F3}
null model	2.72 ± 0.02	0.00 ± 0.00	NA	0.00 ± 0.00	0.00 ± 0.00
LR	2.17 ± 0.01	0.60 ± 0.01	0.60 ± 0.01	0.36 ± 0.01	0.36 ± 0.01
kNN	1.65 ± 0.05	0.80 ± 0.02	0.79 ± 0.02	0.63 ± 0.02	0.63 ± 0.02
lSVR	2.19 ± 0.02	0.60 ± 0.01	0.59 ± 0.01	0.36 ± 0.01	0.36 ± 0.01
SVR	1.74 ± 0.01	0.77 ± 0.00	0.77 ± 0.00	0.59 ± 0.01	0.59 ± 0.01
DNN	1.86 ± 0.03	0.73 ± 0.01	0.73 ± 0.01	0.53 ± 0.02	0.53 ± 0.02
RF	0.70 ± 0.01	0.98 ± 0.00	0.98 ± 0.00	0.93 ± 0.00	0.93 ± 0.00
eRF	0.00 ± 0.00	1.00 ± 0.00	1.00 ± 0.00	1.00 ± 0.00	1.00 ± 0.00

Table SI 5: Comparison of the performance of different models on the PDBbind validation set using five different metrics. Results for the different random test set splits and cross-validation folds were averaged and the mean and standard deviation are reported. The RMSE is given in kcal/mol. NA: Not applicable

model	RMSE	r	ρ	R^2	Q^2_{F3}
null model	2.72 ± 0.07	0.00 ± 0.00	NA	-0.00 ± 0.00	-0.00 ± 0.06
LR	2.19 ± 0.06	0.59 ± 0.02	0.59 ± 0.03	0.35 ± 0.03	0.35 ± 0.04
kNN	2.02 ± 0.05	0.68 ± 0.02	0.67 ± 0.02	0.45 ± 0.03	0.45 ± 0.03
lSVR	2.20 ± 0.07	0.59 ± 0.02	0.59 ± 0.03	0.35 ± 0.03	0.35 ± 0.04
SVR	2.03 ± 0.06	0.67 ± 0.02	0.66 ± 0.02	0.45 ± 0.03	0.45 ± 0.04
DNN	2.02 ± 0.05	0.67 ± 0.02	0.66 ± 0.02	0.45 ± 0.03	0.45 ± 0.03
RF	1.88 ± 0.05	0.73 ± 0.02	0.72 ± 0.02	0.52 ± 0.02	0.52 ± 0.03
eRF	1.85 ± 0.05	0.74 ± 0.02	0.73 ± 0.02	0.54 ± 0.02	0.54 ± 0.03

Table SI 6: Performance of the model for the individual protein targets provided by Wang et al.[2]

data set	N	RMSE	r	ρ	R^2	Q^2_{F3}
BACE	36	0.96 ± 0.06	-0.1 ± 0.1	-0.1 ± 0.1	-0.5 ± 0.2	0.87 ± 0.02
CDK2	16	1.8 ± 0.1	-0.2 ± 0.2	0.0 ± 0.2	-1.4 ± 0.3	0.55 ± 0.05
Mcl-1	67	1.86 ± 0.08	0.70 ± 0.02	0.64 ± 0.07	-0.5 ± 0.1	0.53 ± 0.04
PTP1B	48	1.22 ± 0.09	0.78 ± 0.02	0.72 ± 0.03	0.48 ± 0.08	0.80 ± 0.03
TYK2	71	1.13 ± 0.03	0.67 ± 0.01	0.54 ± 0.03	0.39 ± 0.03	0.83 ± 0.01
Thrombin	11	1.7 ± 0.1	0.4 ± 0.1	0.34 ± 0.09	-2.6 ± 0.5	0.61 ± 0.06
p38	34	0.98 ± 0.04	0.3 ± 0.1	0.3 ± 0.1	0.03 ± 0.07	0.87 ± 0.01

Table SI 7: Performance of the model for the individual protein targets in the CSAR 2012 data set.[4]

data set	N	RMSE	r	ρ	R^2	Q^2_{F3}
CDK2	25	1.9 ± 0.1	0.50 ± 0.05	0.50 ± 0.07	-1.8 ± 0.3	0.53 ± 0.06
CDK2-Cyclin A	23	1.09 ± 0.09	0.65 ± 0.03	0.47 ± 0.04	-0.1 ± 0.2	0.84 ± 0.03
CHK1	110	1.71 ± 0.04	0.12 ± 0.03	0.05 ± 0.03	-0.33 ± 0.06	0.60 ± 0.02
ERK2	298	1.34 ± 0.02	0.36 ± 0.05	0.33 ± 0.05	0.08 ± 0.03	0.76 ± 0.01
LpxC	20	2.1 ± 0.1	0.38 ± 0.03	0.30 ± 0.04	-0.6 ± 0.2	0.39 ± 0.06
Urokinase	35	1.60 ± 0.06	0.46 ± 0.02	0.46 ± 0.03	-0.4 ± 0.1	0.66 ± 0.03

Table SI 8: Performance of the model for the individual protein targets in the CSAR 2014 data set.[5]

data set	N	RMSE	r	ρ	R^2	Q^2_{F3}
HSP90	146	1.81 ± 0.04	0.33 ± 0.02	0.32 ± 0.02	-0.40 ± 0.06	0.56 ± 0.02
SYK	583	1.23 ± 0.04	0.0 ± 0.1	0.0 ± 0.1	-0.10 ± 0.07	0.79 ± 0.01
TrmD	31	1.19 ± 0.06	0.63 ± 0.03	0.46 ± 0.04	0.35 ± 0.07	0.81 ± 0.02

Table SI 9: Enrichment factors for the 102 targets of the DUD-E data set for different fractions of the total data selected.[6] Union describes the combination of all machine learning methods, where redundant molecules are removed.

Target	Fraction (%)	eRF	RF	DNN	kNN	lSVR	SVR	LR	Union
AA2AR	1	1.9	1.7	0.4	1.7	1.2	2.4	2.1	1.4
Active: 844	5	3.0	2.3	1.0	1.4	1.4	1.5	1.6	1.7
Decoys: 32063	10	2.4	2.2	0.9	1.3	1.1	1.2	1.4	1.8
ABL1	1	0.7	0.7	1.7	1.0	4.1	2.7	4.4	2.1
Active: 295	5	0.9	0.5	1.4	1.1	3.7	2.2	4.0	1.9
Decoys: 10885	10	0.8	0.5	1.2	0.9	3.1	1.7	3.2	1.8
ACE	1	3.0	0.6	0.4	3.1	0.5	1.9	0.5	1.0
Active: 803	5	2.4	0.3	1.2	1.5	0.5	0.9	0.4	1.8
Decoys: 17144	10	1.9	0.7	1.4	1.1	0.6	1.1	0.7	1.6
ACES	1	0.3	2.3	0.5	0.6	12.4	18.7	13.1	8.7
Active: 664	5	0.4	1.6	1.8	0.9	6.3	6.3	6.7	5.7
Decoys: 26373	10	0.3	1.2	2.0	0.8	4.1	3.7	4.3	3.8
ADA	1	0.0	0.0	0.4	0.0	0.4	1.2	0.4	0.0
Active: 262	5	0.2	0.2	0.3	0.2	0.7	0.3	0.8	0.3
Decoys: 5472	10	0.3	0.3	0.5	0.4	0.6	0.3	0.6	0.3
ADA17	1	1.4	0.6	4.2	0.7	2.3	0.9	1.9	3.1
Active: 959	5	1.5	1.1	2.1	0.9	1.6	1.0	1.6	1.8
Decoys: 36646	10	1.4	1.1	1.5	1.0	1.5	0.9	1.4	1.6
ADRB1	1	1.7	2.4	7.9	0.2	5.7	14.0	5.9	7.6
Active: 458	5	3.1	3.4	3.1	0.3	2.8	6.2	2.8	4.0
Decoys: 15957	10	2.1	2.3	2.1	0.5	2.4	4.1	2.6	2.9
ADRB2	1	0.7	2.0	2.0	0.7	6.3	13.4	6.7	6.0
Active: 447	5	1.2	1.3	2.3	0.3	2.8	6.3	3.3	4.4

	Fraction (%)	eRF	RF	DNN	kNN	lSVR	SVR	LR	Union
Decoys: 15253	10	0.9	1.4	2.1	0.4	1.8	3.9	2.2	3.0
AKT1	1	0.2	0.2	1.9	1.0	6.9	1.4	7.8	2.6
Active: 423	5	1.8	2.8	2.8	0.6	5.5	3.6	5.8	2.6
Decoys: 16576	10	3.0	3.8	3.7	0.9	4.0	3.1	4.1	2.7
AKT2	1	1.1	1.1	0.0	1.6	7.9	2.1	8.5	3.8
Active: 190	5	2.3	3.5	0.1	1.1	5.6	0.9	5.8	2.7
Decoys: 6952	10	2.1	2.7	0.8	1.1	3.5	1.7	3.6	2.3
ALDR	1	0.0	0.0	0.5	0.5	0.5	0.5	0.5	0.5
Active: 220	5	0.4	0.3	0.5	1.4	0.4	1.3	0.3	0.4
Decoys: 9136	10	0.6	0.5	1.0	1.0	0.3	1.5	0.2	0.7
AMPC	1	0.0	0.0	0.0	0.0	0.0	0.0	0.0	0.0
Active: 62	5	0.0	0.0	0.0	0.0	0.0	0.0	0.0	0.0
Decoys: 2902	10	0.0	0.0	0.0	0.3	0.0	0.0	0.0	0.0
ANDR	1	0.4	1.0	0.4	2.1	1.7	0.8	1.7	1.8
Active: 523	5	1.7	1.5	0.7	2.1	0.6	0.9	0.6	1.3
Decoys: 14503	10	1.8	1.6	0.6	1.5	0.5	0.6	0.5	1.5
AOFB	1	0.6	0.6	1.2	0.6	1.8	0.6	1.8	0.6
Active: 168	5	1.7	1.2	0.4	2.5	2.1	0.2	2.1	1.3
Decoys: 6931	10	1.4	0.9	0.5	2.5	2.0	0.4	2.0	1.3
BACE1	1	0.2	0.0	0.6	4.3	1.2	1.2	0.8	2.5
Active: 485	5	1.0	0.8	0.9	2.1	1.1	1.2	1.1	1.7
Decoys: 18221	10	1.2	0.8	0.9	1.6	1.3	1.3	1.4	1.3
BRAF	1	0.0	4.0	6.4	1.2	2.8	6.8	3.2	2.4
Active: 251	5	2.0	2.1	3.5	1.0	2.6	4.7	2.6	3.0
Decoys: 10098	10	2.1	2.0	2.2	1.6	2.3	3.3	2.4	2.5
CAH2	1	0.8	1.7	3.7	0.6	5.0	1.9	3.7	2.7

	Fraction (%)	eRF	RF	DNN	kNN	lSVR	SVR	LR	Union
Active: 835	5	0.7	1.1	2.2	0.5	2.7	1.3	2.6	2.1
Decoys: 31710	10	0.6	1.0	1.8	0.6	1.7	1.3	1.7	1.5
CASP3	1	0.3	0.0	0.6	0.0	0.9	0.3	0.9	0.3
Active: 349	5	1.0	0.6	0.9	0.1	0.6	0.7	0.7	0.5
Decoys: 10822	10	1.1	1.0	0.8	0.2	0.8	0.9	0.9	0.7
CDK2	1	1.4	1.8	3.5	0.8	2.1	3.3	2.5	2.7
Active: 798	5	1.6	1.8	1.8	1.2	1.8	2.0	1.9	2.2
Decoys: 28328	10	1.4	1.6	1.8	1.2	1.8	1.6	1.9	1.8
COMT	1	0.0	0.0	0.0	0.0	0.0	0.0	0.0	0.0
Active: 86	5	0.0	0.0	0.0	0.0	0.0	0.0	0.0	0.0
Decoys: 3926	10	0.0	0.0	0.2	0.1	0.2	0.1	0.1	0.0
CP2C9	1	1.1	1.7	3.9	1.1	2.2	1.7	2.2	3.4
Active: 183	5	1.5	1.4	2.8	1.4	2.1	2.5	2.2	1.8
Decoys: 7574	10	1.8	1.6	1.8	1.0	1.9	1.9	1.8	1.8
CP3A4	1	0.6	0.3	1.4	0.3	0.6	0.3	0.6	0.3
Active: 359	5	0.6	0.5	0.7	0.9	0.9	0.6	0.9	0.7
Decoys: 11940	10	0.8	0.8	0.7	0.9	1.3	0.4	1.2	0.8
CSF1R	1	3.9	3.5	3.9	0.0	6.3	2.5	6.0	3.5
Active: 286	5	2.7	2.5	3.0	0.8	4.1	3.0	4.8	2.7
Decoys: 12434	10	2.0	2.3	2.4	0.7	3.0	2.4	3.3	2.5
CXCR4	1	0.0	0.0	0.0	0.0	0.0	0.0	0.0	0.0
Active: 122	5	0.2	0.0	0.2	0.8	0.7	0.8	0.7	0.0
Decoys: 3414	10	0.2	0.1	0.8	0.7	0.7	1.4	0.7	0.2
DEF	1	0.0	0.0	0.0	1.9	0.0	0.0	0.0	0.6
Active: 161	5	0.1	0.2	0.0	0.9	0.2	0.0	0.2	0.5

	Fraction (%)	eRF	RF	DNN	kNN	lSVR	SVR	LR	Union
Decoys: 5738	10	0.3	0.4	0.0	1.2	0.3	0.1	0.3	0.4
DHI1	1	1.2	1.0	0.4	1.5	0.4	0.4	0.4	0.8
Active: 519	5	1.2	1.3	1.5	1.2	0.9	0.7	0.8	0.8
Decoys: 19621	10	1.2	1.4	1.4	1.3	0.8	0.9	0.8	1.0
DPP4	1	0.9	0.7	0.2	0.1	0.3	0.9	0.3	0.1
Active: 1079	5	1.0	0.6	0.7	0.9	0.4	0.9	0.3	0.6
Decoys: 41373	10	0.9	0.8	0.7	0.9	0.6	0.8	0.5	0.7
DRD3	1	1.8	9.5	0.9	0.9	8.0	4.9	10.9	6.5
Active: 875	5	2.0	5.2	0.6	0.7	4.8	2.6	5.1	4.6
Decoys: 34188	10	2.0	3.3	0.7	0.7	3.6	1.9	3.9	2.9
DYR	1	0.0	0.2	2.7	1.1	0.5	0.2	0.5	0.9
Active: 566	5	0.4	0.7	1.8	1.6	0.6	0.7	0.8	1.0
Decoys: 17384	10	0.7	0.8	1.3	1.5	0.7	0.9	1.0	1.2
EGFR	1	3.5	4.2	3.9	1.9	7.6	2.8	7.7	4.7
Active: 832	5	2.7	3.0	2.1	1.4	3.8	2.1	3.7	3.5
Decoys: 35442	10	2.4	2.3	1.6	1.2	2.9	2.0	3.0	2.5
ESR1	1	3.5	3.0	2.7	4.0	4.0	2.6	4.0	3.9
Active: 627	5	1.5	1.5	1.2	3.1	2.1	1.0	2.3	2.3
Decoys: 20817	10	1.4	1.4	0.8	2.5	1.8	0.7	1.7	1.9
ESR2	1	3.5	3.5	1.3	1.8	3.0	2.7	2.9	3.2
Active: 595	5	1.8	1.2	1.4	2.4	1.8	1.1	1.8	1.3
Decoys: 20313	10	1.9	1.3	1.0	2.3	1.6	1.0	1.5	1.4
FA10	1	2.5	1.8	0.1	0.9	0.5	0.4	0.3	0.9
Active: 792	5	1.9	1.4	0.9	1.3	0.4	0.8	0.3	0.9
Decoys: 20416	10	1.8	1.7	1.3	1.0	0.8	1.3	0.6	1.0
FA7	1	2.2	2.2	0.0	0.5	0.5	8.8	0.5	2.3

	Fraction (%)	eRF	RF	DNN	kNN	lSVR	SVR	LR	Union
Active: 185	5	2.1	1.8	0.9	1.2	1.6	3.9	2.2	2.7
Decoys: 6302	10	2.3	1.6	1.9	1.7	1.9	3.4	2.2	1.9
FABP4	1	0.0	0.0	0.0	1.8	0.0	0.0	0.0	1.8
Active: 57	5	0.0	0.4	0.0	0.4	0.0	0.0	0.0	0.4
Decoys: 2855	10	0.0	0.2	0.0	0.2	0.0	0.0	0.2	0.2
FAK1	1	0.9	0.9	1.8	1.8	4.4	2.6	5.3	2.7
Active: 114	5	1.4	1.2	3.2	1.8	2.1	2.3	2.5	1.9
Decoys: 5402	10	1.4	1.3	2.3	1.4	1.9	2.3	1.9	2.2
FGFR1	1	1.3	1.3	0.9	1.3	1.3	1.3	1.7	1.8
Active: 242	5	0.9	1.1	0.8	0.9	1.4	0.9	1.3	1.2
Decoys: 494	10	1.0	1.0	0.8	1.0	1.0	0.9	1.1	1.1
FKB1A	1	0.0	0.0	0.0	0.0	0.0	0.0	0.0	0.0
Active: 273	5	0.7	0.7	0.1	1.2	0.3	1.2	0.2	0.2
Decoys: 5832	10	0.8	1.0	0.3	1.6	0.3	1.4	0.3	0.6
FNTA	1	0.1	0.8	0.3	1.1	0.7	1.6	0.7	0.7
Active: 1690	5	0.3	1.0	0.7	0.9	0.7	2.2	0.7	0.8
Decoys: 52048	10	0.4	1.2	1.0	1.4	0.9	1.9	0.8	0.9
FPPS	1	2.8	3.8	0.0	3.3	3.8	0.5	3.8	3.8
Active: 213	5	1.5	0.8	0.0	1.7	1.7	0.6	1.8	1.4
Decoys: 9013	10	0.9	0.6	0.0	1.4	0.9	0.8	1.0	0.8
GCR	1	0.5	0.7	2.0	1.6	0.0	0.9	0.0	0.9
Active: 562	5	0.6	0.6	1.5	0.9	0.3	1.0	0.3	1.0
Decoys: 15185	10	0.8	0.7	1.2	0.6	0.9	1.2	0.8	0.8
GLCM	1	12.3	9.7	0.0	7.4	7.1	11.6	10.0	9.3
Active: 313	5	9.0	9.8	5.1	7.9	5.8	8.3	6.6	7.6

	Fraction (%)	eRF	RF	DNN	kNN	lSVR	SVR	LR	Union
Decoys: 3837	10	5.5	5.5	4.2	5.0	5.5	4.3	5.6	5.7
GRIA2	1	10.5	7.4	5.4	8.5	13.9	14.5	14.5	10.4
Active: 297	5	3.7	4.0	3.1	3.4	3.3	3.6	3.3	3.5
Decoys: 12060	10	2.3	2.3	1.9	2.2	2.3	2.0	2.2	2.2
GRIK1	1	0.7	0.7	2.0	0.0	0.7	0.7	0.7	0.7
Active: 151	5	1.7	1.5	0.9	0.5	1.5	0.5	1.5	0.8
Decoys: 6617	10	1.1	1.2	1.1	0.7	1.0	1.2	0.9	1.1
HDAC2	1	3.8	4.6	4.2	4.6	2.9	2.5	2.9	4.7
Active: 238	5	2.9	3.7	2.5	3.6	4.0	1.9	4.5	3.6
Decoys: 10366	10	2.1	2.3	2.2	2.6	2.9	1.6	3.1	3.0
HDAC8	1	1.7	2.6	2.1	4.3	3.0	2.6	2.6	2.1
Active: 234	5	2.3	2.7	2.2	3.0	2.7	2.1	2.7	2.6
Decoys: 10514	10	2.6	2.5	1.9	1.9	2.1	1.8	1.9	2.0
HIVINT	1	0.5	0.0	0.5	6.2	1.0	1.4	1.0	3.5
Active: 211	5	0.3	0.3	0.5	4.5	0.7	0.3	0.7	2.1
Decoys: 6756	10	0.2	0.3	0.5	3.1	0.6	0.4	0.6	2.4
HIVPR	1	0.1	0.0	2.9	1.5	2.2	0.1	0.9	1.2
Active: 1395	5	0.3	0.3	2.1	1.2	2.0	0.5	1.5	1.7
Decoys: 36277	10	0.6	0.3	1.4	1.2	1.5	0.7	1.3	1.3
HIVRT	1	0.2	0.7	0.5	0.2	1.7	1.0	1.5	1.0
Active: 607	5	0.7	0.8	0.8	0.4	1.0	1.0	1.0	0.7
Decoys: 19133	10	0.7	0.8	0.8	0.8	0.8	1.2	0.8	0.9
HMDH	1	0.0	0.0	1.0	0.0	0.0	1.0	0.0	0.7
Active: 299	5	0.9	0.6	1.5	0.0	0.3	1.9	0.3	0.7
Decoys: 8884	10	1.2	0.7	1.6	0.6	0.7	1.6	0.6	0.8
HS90A	1	0.0	0.0	4.9	0.0	0.8	0.8	0.8	1.7

	Fraction (%)	eRF	RF	DNN	kNN	lSVR	SVR	LR	Union
Active: 125	5	0.3	0.3	2.9	0.2	0.5	2.1	0.5	1.4
Decoys: 4942	10	0.2	0.2	2.1	0.2	0.4	1.6	0.3	1.3
HXK4	1	0.0	0.0	3.2	3.2	0.0	0.8	0.0	0.9
Active: 127	5	0.0	0.0	3.9	2.1	0.5	1.9	0.5	1.6
Decoys: 4803	10	0.2	0.2	3.0	2.4	0.9	1.7	1.1	1.9
IGF1R	1	4.9	0.4	5.8	3.1	5.8	3.1	7.5	6.9
Active: 226	5	2.0	1.9	2.0	2.2	4.3	1.9	4.5	4.1
Decoys: 9407	10	1.8	2.0	1.8	2.1	3.5	1.9	3.7	2.7
INHA	1	2.9	1.5	1.5	2.9	0.0	2.9	0.0	1.5
Active: 71	5	2.3	2.0	0.6	2.0	1.7	0.6	1.7	2.0
Decoys: 2318	10	1.6	1.7	0.8	1.8	1.7	1.1	2.1	1.3
ITAL	1	0.4	1.3	0.4	1.7	0.0	0.4	0.0	0.9
Active: 233	5	1.1	1.2	0.1	1.1	0.9	0.1	0.9	0.5
Decoys: 8689	10	1.2	1.1	0.2	0.9	0.9	0.1	0.9	0.7
JAK2	1	3.3	1.3	2.0	0.7	2.0	1.3	2.0	1.3
Active: 153	5	2.6	2.5	1.8	1.6	1.8	2.1	1.6	1.6
Decoys: 6590	10	1.7	1.8	1.5	1.6	1.6	1.8	1.8	1.5
KIF11	1	0.0	0.0	0.0	0.5	0.0	0.0	0.0	0.0
Active: 197	5	0.2	0.3	0.0	0.2	0.1	0.1	0.1	0.2
Decoys: 6912	10	0.3	0.5	0.8	0.2	0.5	0.4	0.6	0.2
KIT	1	8.0	4.8	3.2	0.0	7.2	4.4	10.8	5.7
Active: 252	5	4.3	4.9	1.9	0.4	4.4	2.9	5.6	3.7
Decoys: 10609	10	3.1	3.0	1.8	0.6	3.7	2.5	4.0	3.3
KITH	1	0.0	0.0	0.0	0.0	0.0	0.0	0.0	0.0
Active: 132	5	0.0	0.0	0.0	0.2	0.0	0.0	0.0	0.0

	Fraction (%)	eRF	RF	DNN	kNN	lSVR	SVR	LR	Union
Decoys: 2866	10	0.1	0.0	0.2	0.2	0.2	0.0	0.1	0.0
KPCB	1	1.2	1.2	0.0	0.0	1.6	0.4	2.0	1.2
Active: 248	5	1.2	1.5	0.0	0.9	1.0	0.2	1.3	1.1
Decoys: 8844	10	0.7	1.2	0.2	0.8	1.2	0.2	1.4	1.0
LCK	1	2.9	3.8	4.0	2.1	7.2	3.8	9.1	5.9
Active: 683	5	1.8	1.6	1.8	1.3	4.5	2.1	5.1	3.6
Decoys: 27856	10	1.7	1.6	1.6	1.3	3.5	1.7	3.6	2.9
LKHA4	1	3.7	4.5	0.0	0.4	1.6	6.2	2.1	5.8
Active: 244	5	1.5	1.3	0.5	0.8	1.2	3.7	1.5	1.8
Decoys: 9477	10	1.0	1.2	0.9	0.9	2.4	2.1	2.4	1.8
MAPK2	1	2.9	3.4	0.5	2.0	3.9	0.5	5.9	2.0
Active: 206	5	2.5	1.9	1.1	1.7	2.2	1.3	2.7	2.0
Decoys: 6244	10	2.0	1.7	1.0	1.5	1.9	1.7	2.2	1.9
MCR	1	0.0	0.0	0.0	1.0	0.0	0.0	0.0	0.5
Active: 193	5	0.0	0.1	0.1	0.9	0.0	0.1	0.0	0.2
Decoys: 5240	10	0.1	0.1	0.3	1.6	0.1	0.3	0.1	0.3
MET	1	5.0	2.1	1.2	1.7	2.9	2.1	3.3	2.1
Active: 244	5	5.2	4.2	0.7	0.8	4.1	1.3	4.2	2.3
Decoys: 11433	10	4.1	4.2	0.9	0.8	2.7	1.4	2.7	2.3
MK01	1	4.4	5.1	0.7	0.7	0.7	0.0	0.7	1.6
Active: 139	5	2.2	1.7	1.9	1.4	1.3	1.6	1.7	2.0
Decoys: 4627	10	1.6	2.2	1.6	1.6	1.4	1.3	1.4	1.7
MK10	1	5.9	5.4	0.5	2.7	2.7	0.5	4.3	0.6
Active: 186	5	2.0	2.0	1.3	2.0	2.8	0.4	2.9	2.5
Decoys: 6714	10	1.7	1.6	1.3	1.6	2.4	0.5	2.4	1.7
MK14	1	2.3	3.1	3.5	2.8	3.4	2.6	3.0	2.8

	Fraction (%)	eRF	RF	DNN	kNN	lSVR	SVR	LR	Union
Active: 915	5	1.5	1.5	2.5	1.5	2.5	1.7	2.6	2.5
Decoys: 36432	10	1.3	1.2	2.0	1.4	2.0	1.4	2.0	2.0
MMP13	1	0.7	0.6	4.1	1.7	2.3	2.7	1.8	2.9
Active: 1038	5	1.1	1.0	2.1	0.6	1.7	1.7	1.5	2.0
Decoys: 38008	10	1.0	1.0	1.9	0.9	1.7	1.4	1.6	1.6
MP2K1	1	0.8	1.7	5.0	0.0	7.1	4.6	5.8	3.4
Active: 242	5	2.4	2.6	3.4	1.4	2.6	2.8	2.2	3.0
Decoys: 8240	10	2.0	1.9	2.3	1.1	2.4	2.0	2.5	2.4
NOS1	1	5.6	7.7	5.1	0.4	9.8	8.1	10.3	6.3
Active: 234	5	4.5	3.8	2.6	3.5	3.4	3.0	4.3	3.0
Decoys: 8073	10	2.5	2.5	2.4	3.0	2.6	2.7	2.6	2.3
NRAM	1	0.5	0.0	0.0	2.7	0.0	0.0	0.0	1.4
Active: 222	5	1.4	0.5	0.0	2.1	0.2	0.1	0.2	0.8
Decoys: 6227	10	1.7	0.3	0.0	1.7	0.2	0.2	0.2	1.2
PA2GA	1	1.6	0.8	1.6	0.8	6.4	0.0	5.6	1.6
Active: 127	5	0.9	0.6	1.1	0.5	2.5	1.1	2.7	1.8
Decoys: 5216	10	0.9	1.1	1.3	0.3	1.8	1.0	1.9	1.5
PARP1	1	0.8	0.7	0.1	2.0	1.2	0.3	1.5	0.7
Active: 742	5	1.2	1.3	0.3	1.6	1.0	0.2	1.1	1.1
Decoys: 30428	10	1.3	1.3	0.5	1.6	1.2	0.4	1.5	1.1
PDE5A	1	1.3	0.7	1.3	1.3	0.7	1.4	0.7	1.9
Active: 706	5	1.4	1.2	1.6	1.0	1.6	1.4	1.7	1.0
Decoys: 27826	10	1.2	1.5	1.6	1.1	1.6	1.4	1.6	1.2
PGH1	1	0.8	0.4	0.8	0.4	0.8	0.4	0.8	1.2
Active: 251	5	0.8	0.6	0.8	0.6	0.7	1.0	0.6	0.7

	Fraction (%)	eRF	RF	DNN	kNN	lSVR	SVR	LR	Union
Decoys: 10942	10	0.7	0.7	1.1	0.6	0.6	1.0	0.6	0.9
PGH2	1	0.6	0.8	0.8	1.1	0.8	0.9	0.8	1.1
Active: 531	5	0.5	0.6	0.9	0.7	0.9	1.0	0.8	0.8
Decoys: 23405	10	0.6	0.8	0.9	0.8	1.0	0.9	1.0	0.8
PLK1	1	1.3	1.9	0.6	1.9	7.1	0.6	5.2	4.7
Active: 155	5	0.4	1.0	1.2	0.9	3.4	0.6	3.2	2.3
Decoys: 6879	10	0.8	1.1	1.3	1.0	3.0	1.2	2.5	1.9
PNPH	1	9.5	7.8	0.0	2.2	0.0	0.4	0.0	3.6
Active: 233	5	3.8	3.7	0.9	3.0	0.6	0.5	0.6	2.9
Decoys: 7016	10	2.4	2.4	1.2	2.1	0.3	0.8	0.4	2.3
PPARA	1	1.5	0.7	0.2	0.4	1.1	0.0	0.9	0.6
Active: 544	5	1.1	1.8	0.5	0.6	2.6	0.1	2.7	0.9
Decoys: 19831	10	1.0	2.2	0.6	1.0	2.4	0.2	2.5	0.9
PPARD	1	0.3	0.3	0.0	0.0	0.3	0.0	0.3	0.0
Active: 288	5	1.0	0.8	0.1	0.2	1.9	0.2	1.9	0.2
Decoys: 13232	10	1.0	1.8	0.1	0.2	2.2	0.2	2.2	0.3
PPARG	1	1.8	1.9	1.0	0.7	1.0	1.4	1.4	0.8
Active: 723	5	2.4	1.2	1.9	0.9	2.8	0.9	2.8	1.6
Decoys: 25867	10	1.9	1.0	2.0	1.1	2.6	0.6	2.7	1.6
PRGR	1	0.5	0.5	0.0	2.3	0.0	0.7	0.0	0.7
Active: 444	5	0.9	0.7	0.1	1.4	0.5	0.5	0.5	0.9
Decoys: 15814	10	1.4	1.1	0.7	1.4	0.6	0.6	0.6	0.9
PTN1	1	10.7	10.3	5.4	1.3	1.8	1.8	2.7	7.2
Active: 225	5	4.3	4.5	2.6	3.3	2.8	2.6	1.6	3.1
Decoys: 7433	10	3.4	2.9	2.4	2.9	2.6	1.9	2.6	3.1
PUR2	1	0.0	0.5	0.0	13.1	0.0	0.0	0.0	3.4

	Fraction (%)	eRF	RF	DNN	kNN	lSVR	SVR	LR	Union
Active: 201	5	0.5	0.2	0.0	8.5	0.0	0.0	0.0	3.6
Decoys: 2725	10	0.4	0.3	0.0	5.5	0.1	0.0	0.2	3.6
PYGM	1	0.0	0.9	0.0	0.0	0.0	0.0	0.0	0.0
Active: 114	5	0.9	0.9	0.9	0.7	0.7	0.9	0.9	0.9
Decoys: 4044	10	0.6	0.9	0.5	1.0	0.4	0.7	0.4	0.6
PYRD	1	0.8	0.0	2.3	0.8	1.5	0.8	0.8	0.0
Active: 134	5	1.2	0.9	1.2	0.4	0.9	0.7	0.9	1.0
Decoys: 6648	10	0.7	1.0	0.8	1.3	1.0	0.6	1.0	0.7
RENI	1	0.0	0.0	0.0	0.5	0.0	5.0	0.0	1.4
Active: 387	5	0.1	0.1	2.4	0.8	0.4	2.6	0.4	1.1
Decoys: 6984	10	0.1	0.2	2.0	0.8	0.6	1.9	0.5	1.3
ROCK1	1	0.0	1.0	1.0	0.0	2.0	1.0	2.0	0.5
Active: 203	5	0.6	0.6	1.7	0.5	1.8	1.2	1.8	0.7
Decoys: 6377	10	0.6	0.6	1.2	0.7	1.0	1.3	1.0	0.9
RXRA	1	1.2	1.2	0.6	0.6	1.2	0.6	1.2	2.5
Active: 162	5	1.7	1.7	1.1	0.5	1.2	1.0	1.1	1.2
Decoys: 7707	10	1.1	1.1	0.6	0.3	0.7	0.6	0.7	1.0
SAHH	1	0.0	0.0	0.0	0.0	0.0	0.0	0.0	0.0
Active: 190	5	0.2	0.3	1.8	0.3	0.0	1.1	0.0	0.4
Decoys: 3483	10	0.4	0.5	2.3	0.2	0.3	2.2	0.3	0.9
SRC	1	6.0	5.3	0.6	1.6	11.0	1.8	11.7	4.4
Active: 831	5	4.1	4.5	1.3	0.8	5.2	2.0	5.7	3.8
Decoys: 34959	10	3.1	3.1	1.2	0.9	3.8	1.8	4.1	2.8
TGFR1	1	0.7	2.1	4.7	0.4	3.9	8.6	4.3	2.9
Active: 281	5	3.7	3.3	4.7	0.5	3.2	3.4	4.0	2.9

	Fraction (%)	eRF	RF	DNN	kNN	lSVR	SVR	LR	Union
Decoys: 8677	10	2.7	3.0	3.0	0.4	3.4	2.8	3.8	2.6
THB	1	1.8	6.0	0.6	1.8	0.6	0.0	0.6	4.2
Active: 168	5	2.0	2.3	0.6	1.7	0.6	0.7	0.4	1.9
Decoys: 7652	10	1.6	1.5	1.4	1.4	1.0	1.7	0.8	1.7
THRB	1	0.9	0.1	0.0	1.5	0.5	0.0	0.3	0.7
Active: 861	5	0.7	0.5	0.5	1.2	1.0	0.8	1.1	0.7
Decoys: 27321	10	0.8	0.7	0.7	1.2	1.2	1.0	1.3	0.9
TRY1	1	0.1	0.5	0.9	0.1	1.1	3.6	1.2	2.0
Active: 758	5	0.6	0.8	1.1	1.6	1.3	1.6	1.3	1.3
Decoys: 26219	10	0.9	1.1	1.2	1.7	1.4	1.5	1.5	1.2
TRYB1	1	7.1	9.5	8.3	4.1	2.4	9.5	3.5	5.9
Active: 171	5	3.3	4.1	4.9	2.1	2.8	5.1	3.7	5.4
Decoys: 7713	10	2.6	2.6	4.0	1.9	2.7	3.7	2.8	3.5
TYSY	1	0.0	0.0	0.3	0.0	0.3	0.7	0.3	0.4
Active: 311	5	0.3	0.1	0.6	0.1	0.8	1.1	0.9	0.3
Decoys: 6883	10	0.4	0.4	0.5	0.2	0.8	1.1	0.7	0.5
UROK	1	0.3	0.0	0.3	0.0	0.0	2.0	0.7	0.0
Active: 306	5	0.9	0.7	1.6	1.2	2.4	2.3	2.8	1.3
Decoys: 9933	10	1.3	1.3	1.6	1.2	1.9	1.9	2.1	1.6
VGFR2	1	1.5	1.9	7.4	0.8	10.6	3.7	11.3	6.0
Active: 620	5	1.4	2.1	3.8	1.3	5.7	3.5	6.1	4.7
Decoys: 25280	10	1.9	2.2	3.0	1.4	4.0	2.9	4.3	3.8
WEE1	1	0.0	0.7	0.0	0.7	8.1	0.0	5.2	2.4
Active: 137	5	0.4	0.1	0.4	0.6	4.7	0.3	4.2	2.2
Decoys: 6234	10	0.4	0.3	0.7	0.4	3.4	0.8	3.3	1.7
XIAP	1	0.0	0.0	0.0	0.0	0.0	0.0	0.0	0.0

	Fraction (%)	eRF	RF	DNN	kNN	lSVR	SVR	LR	Union
Active: 129	5	0.2	0.2	0.0	0.6	0.5	0.0	0.6	0.0
Decoys: 5213	10	0.4	0.1	0.0	0.4	1.1	0.0	1.1	0.4

Supplementary figures

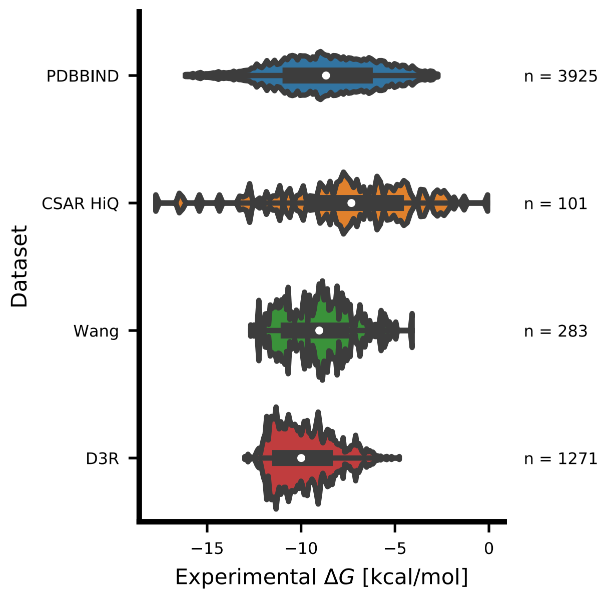

Figure SI 1: Distribution of binding free energy (ΔG) in the protein-ligand complexes in the training and external data sets. D3R contains protein-ligand complexes from both the CSAR 2012 and 2014 releases.[4,5]

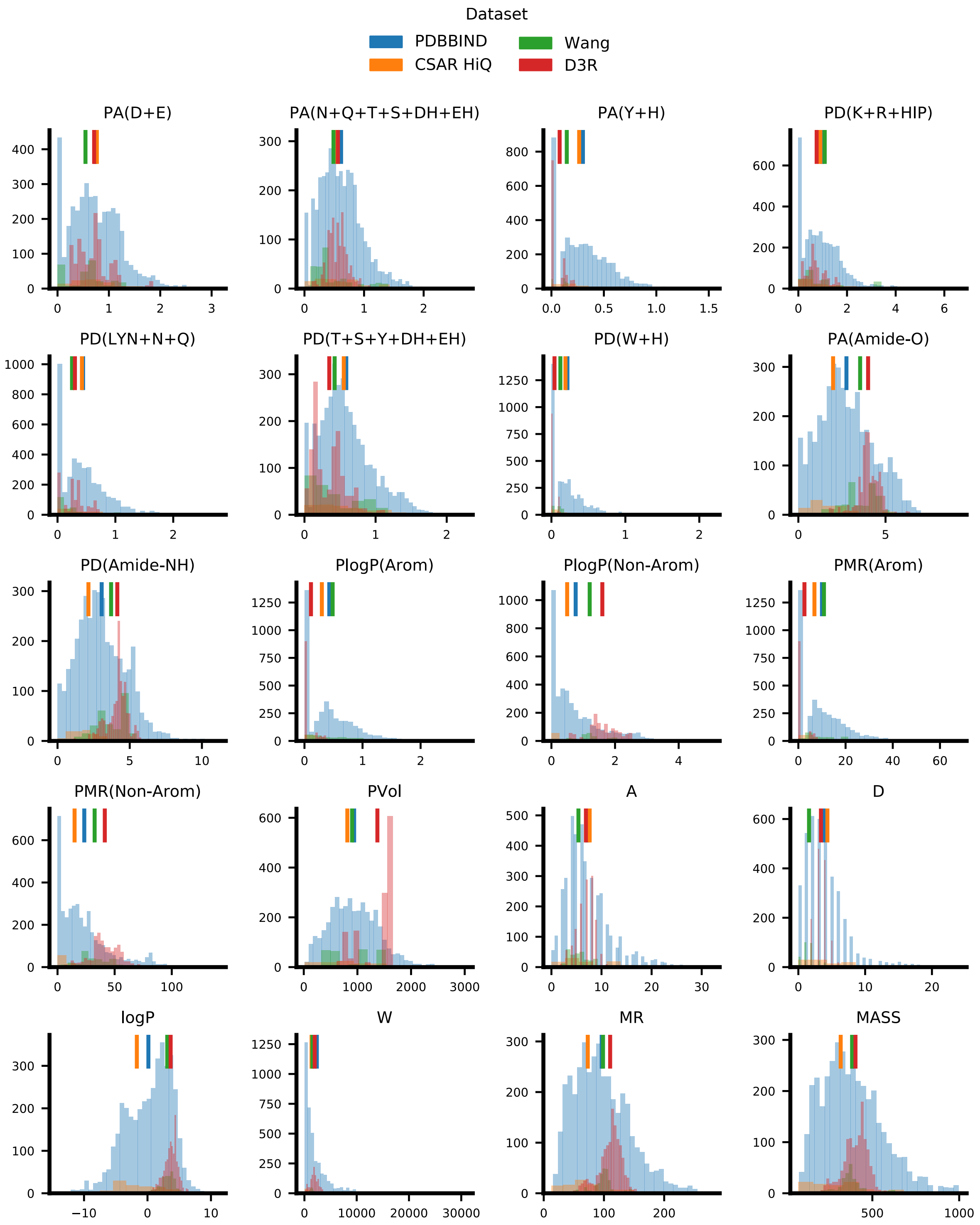

Figure SI 2: Distribution of the protein and ligand descriptor values in the training set and the external test sets shown colored by data set. The vertical lines indicate the mean values for the respective data sets. D3R contains protein-ligand complexes from both the CSAR 2012 and 2014 releases.[4,5]

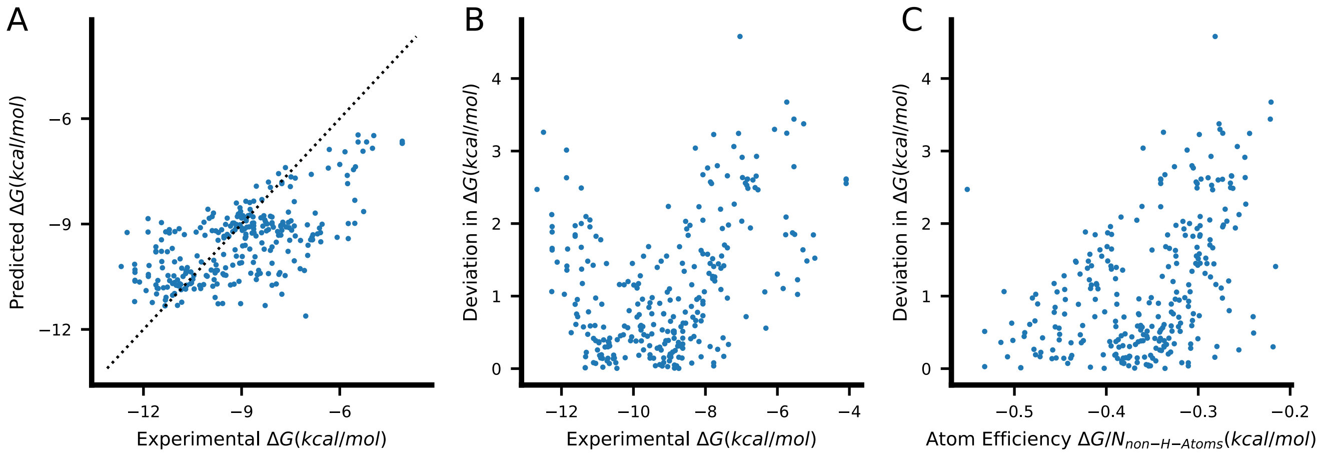

Figure SI 3: Individual predictions on the external data set from Wang et al.[2] (A) Predicted ΔG against experimental ΔG. (B) Error for each prediction against the experimental ΔG. (C) Error for each prediction against the atom efficiency ($\Delta G/N_{non-H-atoms}$).

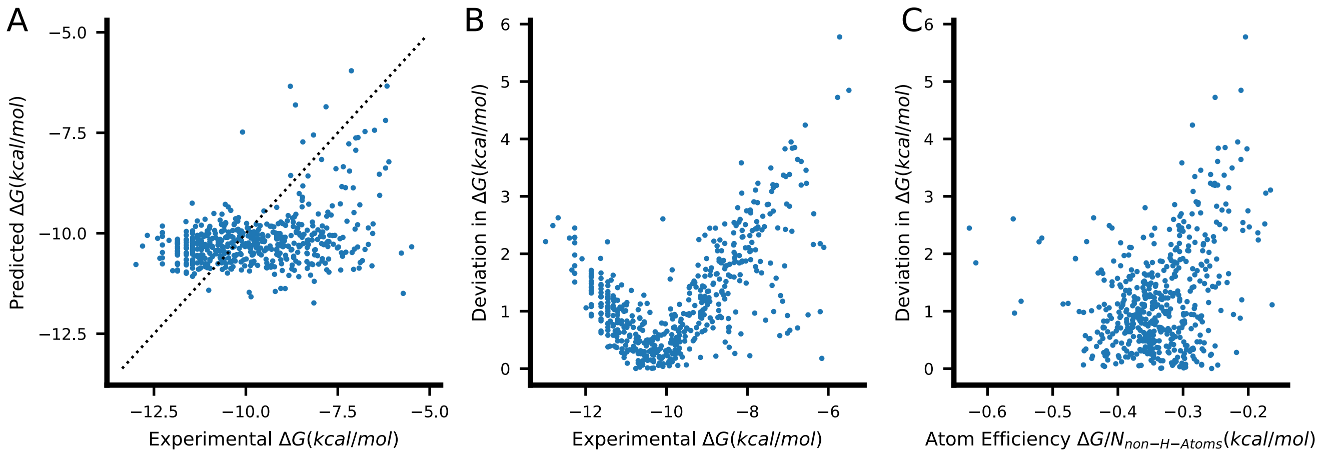

Figure SI 4: Individual predictions on the external CSAR 2012 data set. (A) Predicted ΔG against experimental ΔG. (B) Error for each prediction against the experimental ΔG. (C) Error for each prediction against the atom efficiency ($\Delta G/N_{non-H-atoms}$).

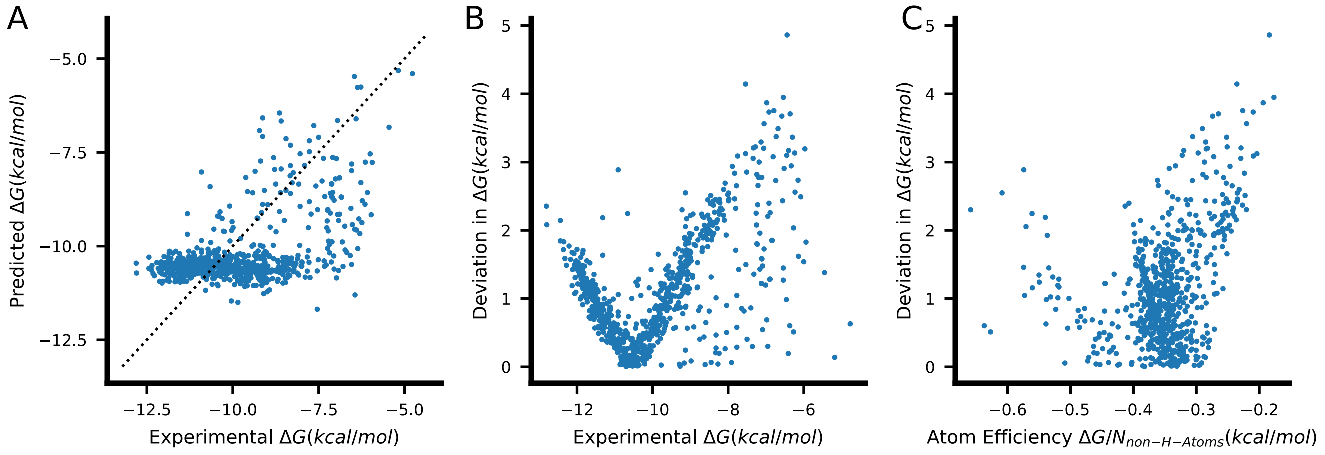

Figure SI 5: Individual predictions on the external CSAR 2014 data set. (A) Predicted ΔG against experimental ΔG. (B) Error for each prediction against the experimental ΔG. (C) Error for each prediction against the atom efficiency ($\Delta G/N_{non-H-atoms}$).

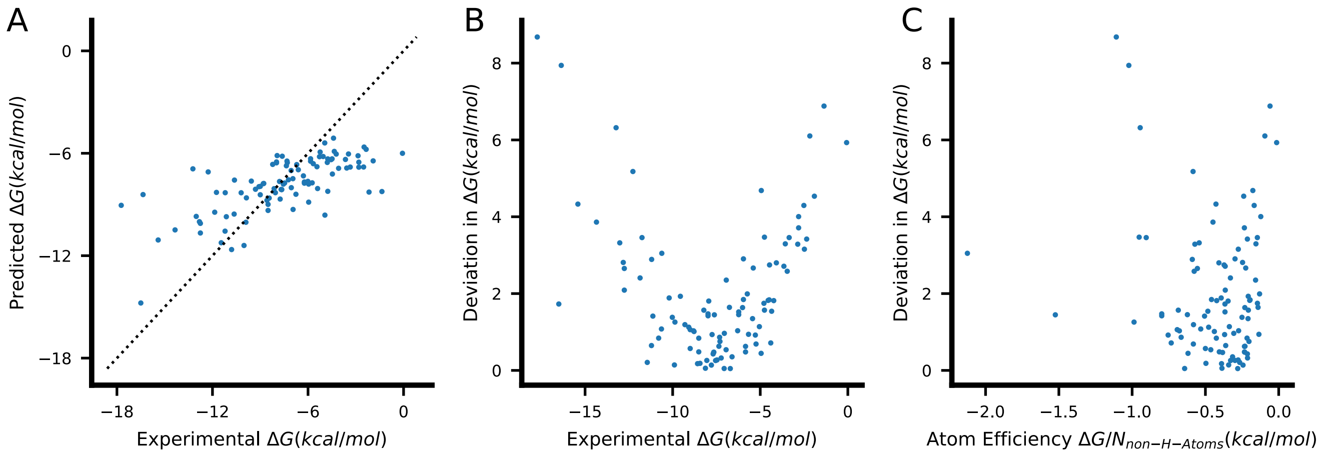

Figure SI 6: Individual predictions on the external CSAR-NSR HiQ data set. (A) Predicted ΔG against experimental ΔG. (B) Error for each prediction against the experimental ΔG. (C) Error for each prediction against the atom efficiency ($\Delta G/N_{non-H-atoms}$).

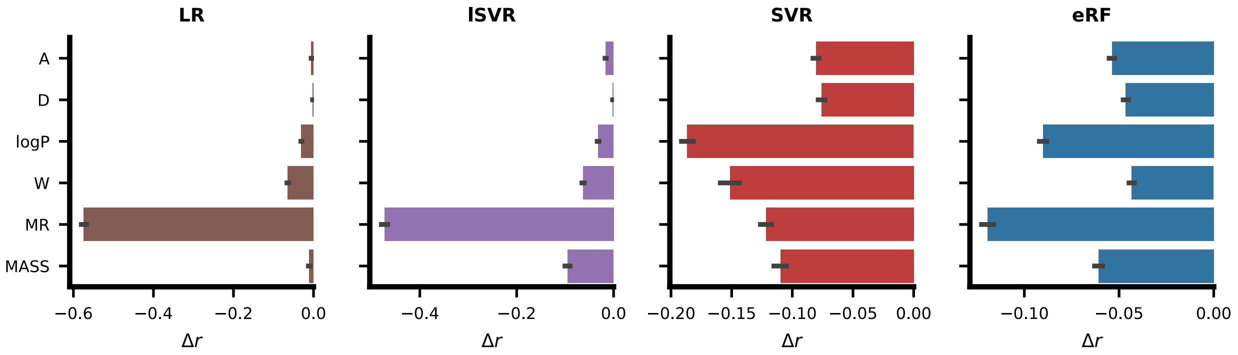

Figure SI 7: Feature importance for models trained with only the ligand descriptors. The feature importance is computed as the average change in Pearson correlation coefficient for five permutations of the respective feature column by shuffling the data (see Methods for details.)

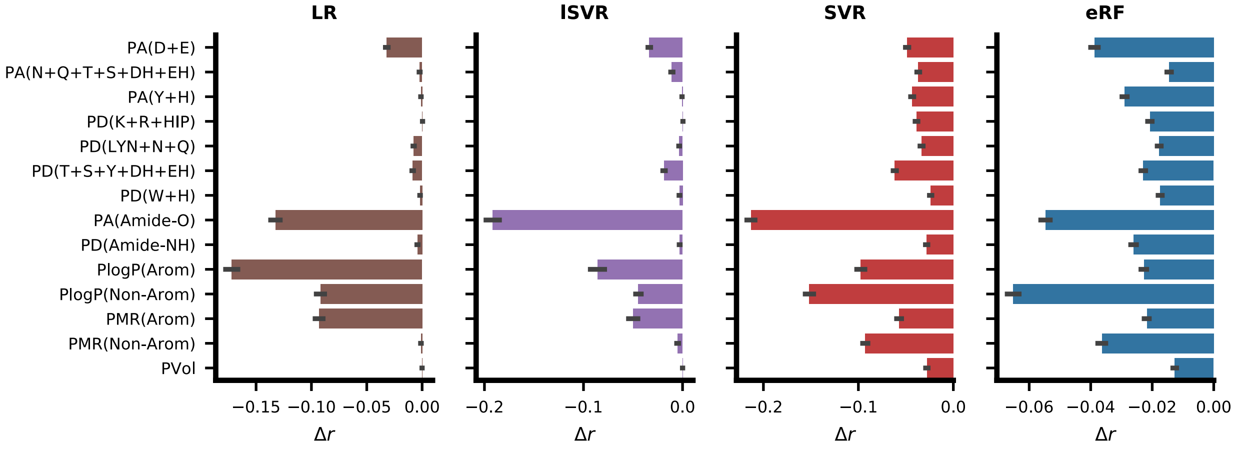

Figure SI 8: Feature importance for models trained with only the protein descriptors. Feature importance is computed as the average change in Pearson correlation coefficient for five permutations of the respective feature column by shuffling the data (see Methods for details).

References

(1) Gathiaka, S.; Liu, S.; Chiu, M.; Yang, H.; Stuckey, J. A.; Kang, Y. N.; Delproposto, J.; Kubish, G.; Dunbar, J. B.; Carlson, H. A.; Burley, S. K.; Walters, W. P.; Amaro, R. E.; Feher, V. A.; Gilson, M. K. D3R grand challenge 2015: Evaluation of protein–ligand pose and affinity predictions. *Journal of Computer-Aided Molecular Design* **2016**, *30*, 651–668, DOI: 10.1007/s10822-016-9946-8.

(2) Wang, L. et al. Accurate and Reliable Prediction of Relative Ligand Binding Potency in Prospective Drug Discovery by Way of a Modern Free-Energy Calculation Protocol and Force Field. *Journal of the American Chemical Society* **2015**, *137*, 2695–2703, DOI: 10.1021/ja512751q.

(3) Wang, R.; Fang, X.; Lu, Y.; Wang, S. The PDBbind Database: Collection of Binding Affinities for Protein-Ligand Complexes with Known Three-Dimensional Structures. *Journal of Medicinal Chemistry* **2004**, *47*, 2977–2980, DOI: 10.1021/jm030580l.

(4) Dunbar, J. B.; Smith, R. D.; Damm-Ganamet, K. L.; Ahmed, A.; Esposito, E. X.; Delproposto, J.; Chinnaswamy, K.; Kang, Y.-N.; Kubish, G.; Gestwicki, J. E.; Stuckey, J. A.; Carlson, H. A. CSAR Data Set Release 2012: Ligands, Affinities, Complexes, and Docking Decoys. *Journal of Chemical Information and Modeling* **2013**, *53*, 1842–1852, DOI: 10.1021/ci4000486.

(5) Carlson, H. A.; Smith, R. D.; Damm-Ganamet, K. L.; Stuckey, J. A.; Ahmed, A.; Convery, M. A.; Somers, D. O.; Kranz, M.; Elkins, P. A.; Cui, G.; Peishoff, C. E.; Lambert, M. H.; Dunbar, J. B. CSAR 2014: A Benchmark Exercise Using Unpublished Data from Pharma. *Journal of Chemical Information and Modeling* **2016**, *56*, 1063–1077, DOI: 10.1021/acs.jcim.5b00523.

(6) Mysinger, M. M.; Carchia, M.; Irwin, J. J.; Shoichet, B. K. Directory of Useful Decoys,

Enhanced (DUD-E): Better Ligands and Decoys for Better Benchmarking. *Journal of Medicinal Chemistry* **2012**, *55*, 6582–6594, DOI: 10.1021/jm300687e.

(7) Mukherjee, G.; Jayaram, B. A rapid identification of hit molecules for target proteins via physico-chemical descriptors. *Physical Chemistry Chemical Physics* **2013**, *15*, 9107, DOI: 10.1039/c3cp44697b.

(8) Drucker, H.; Burges, C. J. C.; Kaufman, L.; Smola, A.; Vapnik, V. Support vector regression machines. Advances in Neural Information Processing Systems 9: Proceedings of the 1996 Conference. 1997; pp 155–161.

(9) Nair, V.; Hinton, G. E. Rectified Linear Units Improve Restricted Boltzmann Machines. Proceedings of the 27th International Conference on International Conference on Machine Learning. USA, 2010; pp 807–814.

(10) Clevert, D. A.; Unterthiner, T.; Hochreiter, S. Fast and accurate deep network learning by exponential linear units (ELUs). 4th International Conference on Learning Representations, ICLR 2016 - Conference Track Proceedings. 2016.

(11) Srivastava, N.; Hinton, G.; Krizhevsky, A.; Salakhutdinov, R. Dropout: A Simple Way to Prevent Neural Networks from Overfitting. *Journal of Machine Learning Research* **2014**, *15*, 1929–1958.

(12) Rumelhart, D. E.; Hinton, G. E.; Williams, R. J. Learning representations by back-propagating errors. *Nature* **1986**, *323*, 533–536, DOI: 10.1038/323533a0.

(13) Breiman, L. Random Forests. *Machine Learning* **2001**, *45*, 5–32, DOI: 10.1023/A:1010933404324.

(14) Geurts, P.; Ernst, D.; Wehenkel, L. Extremely randomized trees. *Machine Learning* **2006**, *63*, 3–42, DOI: 10.1007/s10994-006-6226-1.

(15) Cumming, J. N. et al. Structure based design of iminohydantoin BACE1 inhibitors: Identification of an orally available, centrally active BACE1 inhibitor. *Bioorganic & Medicinal Chemistry Letters* **2012**, *22*, 2444–2449, DOI: https://doi.org/10.1016/j.bmcl.2012.02.013.

(16) Wang, L.; Deng, Y.; Knight, J. L.; Wu, Y.; Kim, B.; Sherman, W.; Shelley, J. C.; Lin, T.; Abel, R. Modeling Local Structural Rearrangements Using FEP/REST: Application to Relative Binding Affinity Predictions of CDK2 Inhibitors. *Journal of Chemical Theory and Computation* **2013**, *9*, 1282–1293, DOI: 10.1021/ct300911a.

(17) Friberg, A.; Vigil, D.; Zhao, B.; Daniels, R. N.; Burke, J. P.; Garcia-Barrantes, P. M.; Camper, D.; Chauder, B. A.; Lee, T.; Olejniczak, E. T.; Fesik, S. W. Discovery of Potent Myeloid Cell Leukemia 1 (Mcl-1) Inhibitors Using Fragment-Based Methods and Structure-Based Design. *Journal of Medicinal Chemistry* **2013**, *56*, 15–30, DOI: 10.1021/jm301448p.

(18) Goldstein, D. M. et al. Discovery of 6-(2,4-difluorophenoxy)-2-[3-hydroxy-1-(2-hydroxyethyl) propylamino]-8-methyl-8 H -pyrido[2,3- d]pyrimidin-7-one (pamapimod) and 6-(2,4-difluorophenoxy)-8-methyl-2-(tetrahydro-2 H -pyran-4-ylamino)pyrido[2,3- d]pyrimidin-7(8 H)-one (R1487). *Journal of Medicinal Chemistry* **2011**, *54*, 2255–2265, DOI: 10.1021/jm101423y.

(19) Wilson, D. P. et al. Structure-Based Optimization of Protein Tyrosine Phosphatase 1B Inhibitors: From the Active Site to the Second Phosphotyrosine Binding Site. *Journal of Medicinal Chemistry* **2007**, *50*, 4681–4698, DOI: 10.1021/jm0702478.

(20) Baum, B.; Mohamed, M.; Zayed, M.; Gerlach, C.; Heine, A.; Hangauer, D.; Klebe, G. More than a Simple Lipophilic Contact: A Detailed Thermodynamic Analysis of Nonbasic Residues in the S1 Pocket of Thrombin. *Journal of Molecular Biology* **2009**, *390*, 56–69, DOI: https://doi.org/10.1016/j.jmb.2009.04.051.

(21) Liang, J. et al. Lead identification of novel and selective TYK2 inhibitors. *European Journal of Medicinal Chemistry* **2013**, *67*, 175–187, DOI: https://doi.org/10.1016/j.ejmech.2013.03.070.

(22) Liang, J. et al. Lead Optimization of a 4-Aminopyridine Benzamide Scaffold To Identify Potent, Selective, and Orally Bioavailable TYK2 Inhibitors. *Journal of Medicinal Chemistry* **2013**, *56*, 4521–4536, DOI: 10.1021/jm400266t.
